# The Presence of Fungal and Parasitic Infections in Substances of Human Origin and Their Transmission via Transfusions and Transplantations: Protocol for Two Systematic Reviews

**DOI:** 10.2196/25674

**Published:** 2021-06-10

**Authors:** Petros C Dinas, Dragoslav Domanovic, Yiannis Koutedakis, Christos Hadjichristodoulou, Ioannis Stefanidis, Kalliope Papadopoulou, Konstantinos Dimas, Konstantinos Perivoliotis, Konstantinos Tepetes, Andreas D Flouris

**Affiliations:** 1 FAME Laboratory Department of Physical Education and Sport Science, University of Thessaly Trikala Greece; 2 European Centre for Disease Prevention and Control Stockholm Sweden; 3 Faculty of Education, Health and Wellbeing Wolverhampton University Wolverhampton United Kingdom; 4 Faculty of Medicine School of Health Sciences, University of Thessaly Larissa Greece; 5 Department of Biochemistry and Biotechnology University of Thessaly Larissa Greece

**Keywords:** fungal and parasitic infections, donor-derived substances, transplantation, transfusion

## Abstract

**Background:**

The European Union Directives stipulate mandatory tests for the presence of any infections in donors and donations of substances of human origin (SoHO). In some circumstances, other pathogens, including fungi and parasites, may also pose a threat to the microbial safety of SoHO.

**Objective:**

The aim of the two systematic reviews is to identify, collect, and evaluate scientific evidence for the presence of fungal and parasitic infections in donors and donations of SoHO, and their transmission via transfusion and transplantation.

**Methods:**

An algorithmic search, one each for fungal and parasitic disease, was applied to 6 scientific databases (PubMed, EMBASE, Web of Science, Scopus, Cochrane Library [trials], and CINAHL). Additionally, manual and algorithmic searches were employed in 15 gray literature databases and 22 scientific organization websites. The criteria for eligibility included peer-reviewed publications and peer-reviewed abstract publications from conference proceedings examining the prevalence, incidence, odds ratios, risk ratios, and risk differences for the presence of fungi and parasites in donors and SoHO donations, and their transmission to recipients. Only studies that scrutinized the donors and donations of human blood, blood components, tissues, cells, and organs were considered eligible. Data extraction from eligible publications will be performed independently by two reviewers. Data synthesis will include a qualitative description of the studies lacking evidence suitable for a meta-analysis and a random or fixed-effect meta-analysis model for quantitative data synthesis.

**Results:**

This is an ongoing study. The systematic reviews are funded by the European Centre for Disease Prevention and Control, and the results are expected to be presented by the end of 2021.

**Conclusions:**

The systematic reviews will provide the basis for developing a risk assessment for fungal and parasitic disease transmission via SoHO.

**Trial Registration:**

PROSPERO International Prospective Register of Systematic Reviews CRD42020160090; https://www.crd.york.ac.uk/prospero/display_record.php?ID=CRD42020160090 ; PROSPERO International Prospective Register of Systematic Reviews CRD42020160110; https://www.crd.york.ac.uk/prospero/display_record.php?ID=CRD42020160110

**International Registered Report Identifier (IRRID):**

DERR1-10.2196/25674

## Introduction

The European Union Directives have set safety interventions to prevent transmission of infections through substances of human origin (SoHO) [1,2]. The safety interventions are essential given a steady increase in transplantations of human tissues and cells [3] and transfusion of blood and blood components [4]. The directives specified mandatory testing of all SoHO donors and donations [1,2,5] for certain viruses and bacteria only. However, additional microbial tests are necessary on certain SoHO donations depending on the epidemiological situation (eg, syphilis, malaria, cytomegalovirus, toxoplasma, Epstein-Barr virus, *Trypanosoma cruzi*) [5].

Surveillance data on the presence and transmission of pathogens via cells, tissue, and blood are currently collected at the national and European Union levels. However, no such data are yet available for organ transplantations [5]. Globally, fungal diseases kill >1.5 million and affect >1 billion people annually, although most of them are preventable [4]. Likewise, parasitic diseases kill >45,000 and affect >23 million people annually worldwide [6], impacting human health and quality of life considerably. Therefore, more studies are needed on the transmission of parasitic diseases via SoHO [3]. Inefficient prevention of the SoHO transmittable diseases poses a significant economic impact on the national health systems. It is, therefore, necessary to conduct a systematic, comprehensive evidence accumulation and data synthesis to assess the risks and identify prevention strategies for fungal and parasitic disease transmission via SoHO to guide the infection risk management. The systematic review process of this protocol aims to identify, collect, and evaluate the evidence of fungal and parasitic infection transmission via SoHO from infectious donors and contaminated donations. The systematic review research questions were identified considering the PICO (Population, Intervention, Comparison, and Outcome) approach [7], including all key elements associated with the two systematic reviews. The two main objectives of this study were to identify the evidence of fungal infections in SoHO donors and donations, and their transmission via transfusion and transplantation, and to identify the evidence of parasitic infections in SoHO donors and donations, and their transmission via transfusion and transplantation.

## Methods

The systematic review protocols are registered with PROSPERO (International Prospective Register of Systematic Reviews); registration numbers: CRD42020160090, CRD42020160110 [8,9]. The PRISMA-P (Preferred Reporting Items for Systematic Reviews and Meta-analyses–Protocols) 2015 checklist used to check the reporting of the current protocol [10] can be found in Multimedia Appendix 1. All authors contributed equally to the systematic reviews’ tasks, drafting the current protocol, and reviewing and approving the final version of the current protocol.

We will employ the FINER (Feasible, Interesting, Novel, Ethical, and Relevant) approach [11] to test the applicability of the research questions.

### FINER Outcome

#### Feasible

A pilot search demonstrated an adequate number of studies for inclusion in the systematic reviews. The algorithmic search in the PubMed database retrieved 2320 and 1895 publications on the relevant fungal and parasitic infections, respectively. Additionally, the technical expertise of the review team, the time allocation, and the available funding guarantee its successful completion.

#### Interesting

The research questions are interesting as the systematic reviews aim at providing vital information on mitigating the risk of fungal and parasitic diseases transmission via SoHO. This is significant because fungal diseases kill >1.5 million and affect >1 billion people annually globally [4], while parasitic diseases kill >45,000 and affect >23 million people annually worldwide [6]. The European Centre for Disease Prevention and Control (ECDC) identified the knowledge gap and invested in addressing the problem [5]. The funding organization (ECDC) approved the current protocol.

#### Novel

The systematic reviews will confirm or reject the earlier findings and produce new findings on the fungal and parasitic infections transmission risks via SoHO donations or contaminations, which would be used to develop future preventive interventions.

#### Ethical

There are no ethical concerns regarding the current systematic review processes, as it will be entirely based on evidence accumulation from earlier studies.

#### Relevant

The research questions are relevant to current scientific knowledge, clinical practices, and health policies.

### Search Strategy

The selection of the information sources was based on the relation of scientific topics to the systematic review research questions and information retrieved from analysis regarding the optimal database combination for biomedical systematic reviews [12]. The review team split the review sources into three broad categories—scientific databases containing peer-reviewed publications, databases with gray literature, and scientific organization websites.

#### Scientific Databases

Scientific databases for peer-reviewed publications included PubMed, EMBASE, Web of Science, Scopus, Cochrane Library (trials), and CINAHL.

#### Gray Literature Databases

Databases for gray literature included JSTOR, OpenGrey, ROAR, ROARMAP, OpenDOAR, GreyNet, British Library, TextRelease, APO, bioRxiv, arXiv, Google Scholar, Infectious Disease Advisor, Healthfinder, and TRIP (Turning Research into Practice).

#### Scientific Organization Websites

Scientific organization websites for identifying peer-reviewed publications, technical reports, and guidelines containing original data included World Health Organization; European Commission; ECDC; Centers for Disease Control and Prevention (CDC); National Health System, United Kingdom; International Foundation for Care; National Health Information Center; Agency for Toxic Substances and Disease Registry; Food and Drug Administration; Indian Health Services; National Center for Chronic Disease Prevention and Health Promotion; National Center for Emerging and Zoonotic Infectious Diseases; National Center for Health Promotion and Disease Prevention, Veterans Health Administration; National Center for Human Immunodeficiency Virus, Sexually Transmitted Disease, and Tuberculosis Prevention, CDC; National Institutes of Health; National Institute of Environmental Health Sciences; Office of Public Health Genomics, CDC; American College of Preventive Medicine; Robert Koch Institute; Australian Department of Health and Aging; National Notifiable Diseases Surveillance System; and ClinicalTrials.gov.

#### Search Procedure

The search strategy was based on the guidelines of the Cochrane Library [7], while relevant PRISMA flowcharts [13] were used to maintain records during the systematic review process. Two independent investigators performed a pilot searching using the PubMed database in September 2019. Following several searching combinations, the review team shaped up two main pilot algorithms (for fungal and parasitic diseases) to be used independently for the scientific databases containing peer-reviewed publications. The algorithms were formed using the Boolean OR, AND, NOT, and several truncations (ie, *). The review team judged both fungal and parasitic diseases pilot algorithms as applicable and appropriate for the official searching procedure. Two members of the review team, PCD and CH, independently conducted the official searching for eligible publications in the selected scientific databases (since their inception to October 2019) for both fungal and parasitic diseases algorithms. These search algorithms were “translated” from one database to another so that the corresponding website search engine could recognize them. ADF and YK confirmed no disagreement between the team members (PCD and CH) in applying the algorithms in all 6 scientific databases. A detailed search history is provided in Multimedia Appendix 1.

The official searching procedure for the gray literature databases and the organizations' websites was accomplished by three members of the review team (PCD, CH, and KP). Following the selection process, a search for the reference lists of systematic reviews, meta-analyses, technical reports, and guidelines relevant to the research questions will be done. Finally, the reference lists of the eligible publications will be screened to identify any research questions–related publications missed out in the initial searching.

### Inclusion Criteria

The inclusion criteria are set for the types of studies, types of participants, and extracted data items.

#### Study Types

The review team identified the following types of studies to be included as eligible in the systematic review: (1) peer-reviewed experimental, epidemiological studies of any methodological design and case reports that examined the prevalence, incidence, odds ratios, risk ratios, and risk differences of the presence of the fungal and parasitic infections in SoHO from an infectious donor or contaminated donation to recipients; (2) peer-reviewed in vitro studies that explored the presence of fungi and parasites in SoHO donors and donations, and transmission via SoHO to humans; (3) technical reports or guidelines by any relevant organizations, where eligible peer-reviewed publications and related original data can be detected; (4) articles from any organizations that investigated disease prevention (eg, guidelines) relevant to the research objectives; (5) outputs in any language; (6) no date limits will be applied in the selection of eligible publications; (7) only peer-reviewed conference proceedings will be eligible from the gray literature, as failure to recognize trials reported in conference proceedings may impose a risk of bias in the effect estimates [14].

#### Participants Type

The type of participants in the eligible studies will be humans. The term “substances of human origin” (ie, SoHO) refers to blood, blood components, tissues, cells, and organs. As a result, therapeutic (plasma derived–medicinal products) and diagnostic products derived from humans will not be included in the systematic review. Therefore, the interventions to be considered will be blood and blood components’ donations and transfusions in humans, and tissue, cell, and organ donations and transplantations in humans.

#### Other Extracted Data Items

The other data items that will be extracted from the databases include the following: (1) population demographics from eligible studies; (2) number of cases and percentages of fungal and parasitic infections in SoHO donors and donations, and their transmission via SoHO donations; (3) the prevalence of fungal and parasitic infections in SoHO donors and donations, and transmission via SoHO donations; (4) odds ratios, risk ratios, and risk differences of the fungal and parasitic infections among SoHO donors and donations, and their transmission risks via SoHO donations between intervention (eg, individual blood transfusion, transplantation, etc) and control groups (eg, healthy individuals, individuals not receiving a blood transfusion, transplantation, etc); (5) associations of donor derived SoHO–related fungal and parasitic disease transmission with demographic characteristics of the population and with any other factor susceptible to the infections; (6) diagnostic methods for the transmissions associated with the relevant SoHO donations (not those related to other exposures); (7) medications received by patients before and during diagnosis; (8) follow-up measures including hospitalizations and deaths; (9) risk factors of the fungi and parasitic infections or contaminations in SoHO donors and donations, and transmissions via SoHO donations; (10) type of infection screening tests in SoHO donors; and (11) country and continent of the population of eligible studies.

### Exclusion Criteria

The exclusion criteria will be: studies not identifying the transmission of the fungal and parasitic diseases via SoHO donations; animal studies; in vitro studies; reviews, systematic reviews, and meta-analyses; letters to editors and opinion papers; theses and dissertations; and gray literature besides the published peer-reviewed conference proceedings relevant to the systematic reviews’ research questions.

### Selection Process

Two members of the review team will select the eligible publications independently. A referee investigator will be asked to decide in case of a disagreement between the two reviewers. The Cohen kappa test will be used to measure the interrater agreement in selecting the eligible publications [15]. A study’s eligibility will be decided by screening the titles and abstracts using the EndNote software files, where the retrieved publications will be saved. The selection review team will ensure the identification and exclusion of retracted publications. The team will also ensure locating the inaccessible eligible publications’ full texts through emails to the lead authors and publication journals. The eligible publications without full texts will be listed in the systematic review with reasons. Finally, for transparency reasons, a complete list of the excluded publications will also be included in the systematic review.

### Data Extraction

An individual data extraction form (Cochrane Library model) [7] will be incorporated for each eligible publication. Two reviewers will extract the data independently from the eligible publications. A referee investigator will make the ultimate decision in case of a disagreement between the reviewers. A priori pilot data extraction will be used to include any missing data that were not initially considered or extracted. The data will be collected as two tables in Excel format, one each for the fungal and the parasitic disease. The data extraction–review team members will contact the corresponding authors via email if the outcome data in the full-text articles are unclear.

### Outcomes and Prioritization

The priority of the outcomes of this systematic review are as follows: (1) intervention type (ie, number of donations, blood or blood components transfusion, type of transplantation); (2) the association of the presence of fungal and parasitic infections in SoHO donors, donations, and their transmission through SoHO donations with any physiological and demographic characteristics of the populations, including aspects such as environmental conditions; (3) the prevalence of the presence of fungal and parasitic infections in SoHO donors, donations, and transmission via SoHO donations; (4) odds ratios, risk ratios, risk differences, and hazard ratios of fungal and parasitic infections or contaminations in SoHO donors, donations, and their transmission via SoHO donations; (5) cause of the infection in donors or the contamination of donation; (6) the type of screening test of infection and the donor types; (7) hospitalizations and deaths; (8) region (country and continent); and (9) diagnostic method.

### Assessment of Methodological Quality

The review team will use the methodological design of each eligible publication to determine the risk of bias assessment. Three appropriate tools are going to be used: the Cochrane Library tool [16] for the risk of bias assessment in randomized controlled trials (RCT); the ROBINS-I tool (Risk of Bias in Nonrandomized Studies–of Interventions) [17] for the risk of bias assessment in non-RCT studies using an intervention, and the RTI item bank tool [18] for the risk of bias assessment in observational studies. Two reviewers will assess the risk of bias in the eligible publications independently, and a referee investigator will decide in case of a disagreement. The Cohen kappa test will be used to measure the interrater agreement in the evaluation results [15]. Finally, the risk of bias assessment results will be extracted in relevant tables and figures according to the Cochrane Library format [7].

### Data Synthesis and Prospective Meta-analysis Methods

The eligible studies with data not suitable for meta-analysis will be summarized into a qualitative description. In the case of the eligible studies with pertinent data for meta-analysis, a random or fixed-effect meta-analysis model will be used to account for heterogeneity due to differences in study populations, types of infections, interventions, study durations, and other factors. All meta-analyses will be conducted using the RevMan 5.3 software (Nordic Cochrane Centre, The Cochrane Collaboration) [19].

The prevalence for meta-analysis will be calculated using the following formula [7]


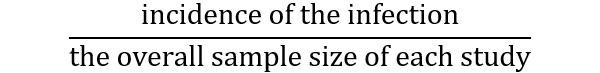


Standard errors for the meta-analysis will be calculated using this formula [7]


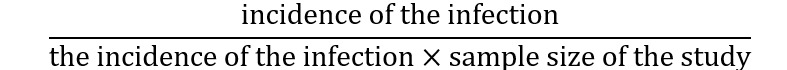


Standard errors will then be used for weighted proportions, and the RevMan 5.3 software [19] will be used to generate the forest and funnel plots. The funnel plots will only be generated for those meta-analyses that include more than 10 studies [7]. The odds ratios, risk ratios, and risk differences for meta-analyses will be calculated using a dichotomous, inverse variance method, referring to infection incidences of the individuals with an intervention (ie, transfusion, transplantation) against infection incidences of individuals without any interventions. The weighted proportions of such meta-analyses will be calculated based on each study’s sample size. The time-to-event outcomes (hazard ratio) for a meta-analysis will be calculated using the generic inverse variance, or an O and E variance fixed effect model [7].

The 95% CI and heterogeneity between studies will be evaluated using the *I*^2^ statistic. The results for heterogeneity will be considered statistically significant at *P*<.10, while the *I*^2^ index interpretations will be made based on earlier guidelines [7]. Small study effects, potentially caused by publication bias, will be assessed using the funnel plots [7]. The risk of bias assessments will be incorporated in data synthesis and used for the forest plots [7]. Finally, the standardized mean difference (SMD) (ie, the difference in mean outcomes between groups/standard deviation of outcomes among participants) will be used for the meta-analysis studies that assess the same outcome using different measurement scales [7].

### Meta-bias Assessment

The reporting of the eligible publications will be checked independently by two review team members, while a referee will make the final decision in case of a discrepancy between them. The interrater agreement in the evaluation results will be tested using the Cohen kappa test [15]. The 25-item checklist of the CONSORT (Consolidated Standards of Reporting Trials) [20] will be adopted for the eligible RCTs. The 22-item STROBE checklist (Strengthening the Reporting of Observational studies in Epidemiology) [21] will be employed for the eligible observational studies and published conference proceedings. A score will be calculated for each eligible study following a previous methodology [22]. The reporting scores of the eligible studies will not be used for assessing the methodological quality of the eligible studies; however, they will be used for measuring the reporting quality as a risk factor for the critical appraisal of eligible papers due to missing information [23].

### Confidence in Cumulative Evidence

Two reviewers will independently appraise the implications and applicability of the findings of the systematic reviews using the GRADE (Grading of Recommendations Assessment, Development, and Evaluation) analysis [24]. The GRADE analysis rates the quality of the best available evidence, which can be used for developing healthcare recommendations and guidelines. The four-level GRADE ratings classify the quality of evidence as high, moderate, low, and very low. The low-quality evidence characteristics include an observational study, risk of bias, inconsistent results, indirectness, imprecision, and publication bias. The high-quality evidence characteristics include an RCT study, a large magnitude of effect, demonstration of a dose–response relationship, and if residual confounding factors that are plausibly expected to reduce or increase the demonstrated effect do not actually reduce or increase it. A critical appraisal of the current systematic review process will be performed using the AMSTAR (A Measurement Tool to Assess Systematic Reviews) checklist [25], a critical appraisal tool for assessing the quality of systematic reviews.

## Results

This is an ongoing study. The systematic reviews are funded by the ECDC, and the results are expected to be announced by the end of 2021.

## Discussion

### Overview

The two systematic reviews aim to identify the scientific evidence on transmission risks of fungal and parasitic diseases via SoHO globally. The retrieved evidence would assist an evidence-based risk assessment of the fungal and parasitic transmission through SoHO donations and evaluating the available prevention strategies within the European Union. The study results will be used to create an evidence pool containing the geographical data for transmission risks, genetic, physiological, and demographic characteristics of the infected populations, the infection cause and the type of screening tests for donors, and diagnostic methods of the infections. It is also possible that a meta-analysis of these data will be incorporated, which will further strengthen the evidence-based risk assessment approach.

### Strengths and Limitations

The systematic review process has many strengths. The protocol followed the PRISMA-P guidelines. The searching procedure used robust algorithms with standardized indexing terms to retrieve records that had different words to describe the same concept and information beyond the words in the title and abstract [7]. We will use well-established tools [16,18] to evaluate the included studies. Additionally, to reduce bias, two investigators will work independently on screening the data for eligibility, risk of bias assessment, data extraction, and the CONSORT and STROBE scores. The search procedure of the systematic reviews had no restrictions regarding the date of publication, study design, and language. The GRADE analysis will allow an excellent evaluation of the quality of the outcomes.

The systematic review process has limitations too. We excluded gray literature, incorporating a publication bias. Nevertheless, the inclusion of gray literature may itself introduce bias, and one reason to include the gray literature is the absence of peer-review sources [7]. Another limitation that we foresee is that we have accepted the peer-reviewed in vitro studies as eligible, even though it is scarce to identify such studies. We have included these studies to increase the transparency and validity of our systematic review process.

### Conclusions

These systematic reviews will form the basis for developing a risk assessment of fungal and parasitic disease transmission via SoHO.

## Appendix

Multimedia Appendix 1 PRISMA-P checklist and searches.

## Abbreviations

AMSTAR: A Measurement Tool to Assess Systematic Reviews
CONSORT: Consolidated Standards of Reporting Trials
ECDC: European Centre for Disease Prevention and Control
FINER: Feasible, Interesting, Novel, Ethical, and Relevant
GRADE: Grading of Recommendations Assessment, Development, and Evaluation
PICO: Population, Intervention, Comparison, and Outcome
PRISMA-P: Preferred Reporting Items for Systematic Reviews and Meta-analyses–Protocols
PROSPERO: International Prospective Register of Systematic Reviews
RCT: randomized controlled trial
SMD: standardized mean difference
SoHO: substances of human origin
STROBE: Strengthening the Reporting of Observational Studies in Epidemiology
TRIP: Turning Research Into Practice
