# Supplementary material for: The Presence of Fungal and Parasitic Infections in Substances of Human Origin and Their Transmission via Transfusions and Transplantations: Protocol for Two Systematic Reviews
Source: JMIR Res Protoc. 2021 Jun 10;10(6):e25674. doi: 10.2196/25674 (PMC8262548; doi:10.2196/25674)
Supplement: Multimedia Appendix 1 [file resprot_v10i6e25674_app1.docx]

**PRISMA-P 2015 Checklist**

| **Section/topic** | **#** | **Checklist item** | **Information reported** | | **Line number(s)** |
| --- | --- | --- | --- | --- | --- |
|  |  |  | **Yes** | **No** |  |
| **ADMINISTRATIVE INFORMATION** | | | | | |
| **Title** | | | | | |
| Identification | 1a | Identify the report as a protocol of a systematic review |  |  | 1 |
| Update | 1b | If the protocol is for an update of a previous systematic review, identify as such |  |  |  |
| **Registration** | 2 | If registered, provide the name of the registry (e.g., PROSPERO) and registration number in the Abstract |  |  | 58-61 |
| **Authors** | | | | | |
| Contact | 3a | Provide name, institutional affiliation, and e-mail address of all protocol authors; provide physical mailing address of corresponding author |  |  | 5-23 |
| Contributions | 3b | Describe contributions of protocol authors and identify the guarantor of the review |  |  | 407-408 |
| **Amendments** | 4 | If the protocol represents an amendment of a previously completed or published protocol, identify as such and list changes; otherwise, state plan for documenting important protocol amendments |  |  |  |
| **Support** | | | | | |
| Sources | 5a | Indicate sources of financial or other support for the review |  |  | 403-405 |
| Sponsor | 5b | Provide name for the review funder and/or sponsor |  |  | 403 |
| Role of sponsor/funder | 5c | Describe roles of funder(s), sponsor(s), and/or institution(s), if any, in developing the protocol |  |  | 404-405 |
| **INTRODUCTION** | | | | | |
| **Rationale** | 6 | Describe the rationale for the review in the context of what is already known |  |  | 72-97 |
| **Objectives** | 7 | Provide an explicit statement of the question(s) the review will address with reference to participants, interventions, comparators, and outcomes (PICO) |  |  | 98-101 |
| **METHODS** | | | | | |
| **Eligibility criteria** | 8 | Specify the study characteristics (e.g., PICO, study design, setting, time frame) and report characteristics (e.g., years considered, language, publication status) to be used as criteria for eligibility for the review |  |  | 186-239 |
| **Information sources** | 9 | Describe all intended information sources (e.g., electronic databases, contact with study authors, trial registers, or other grey literature sources) with planned dates of coverage |  |  | 140-160 |
| **Search strategy** | 10 | Present draft of search strategy to be used for at least one electronic database, including planned limits, such that it could be repeated |  |  | 135-183 & supplement |
| ***STUDY RECORDS*** | | | | | |
| Data management | 11a | Describe the mechanism(s) that will be used to manage records and data throughout the review |  |  | 256-266 |
| Selection process | 11b | State the process that will be used for selecting studies (e.g., two independent reviewers) through each phase of the review (i.e., screening, eligibility, and inclusion in meta-analysis) |  |  | 241-254 |
| Data collection process | 11c | Describe planned method of extracting data from reports (e.g., piloting forms, done independently, in duplicate), any processes for obtaining and confirming data from investigators |  |  | 256-266 |
| **Data items** | 12 | List and define all variables for which data will be sought (e.g., PICO items, funding sources), any pre-planned data assumptions and simplifications |  |  | 210-231 |
| **Outcomes and prioritization** | 13 | List and define all outcomes for which data will be sought, including prioritization of main and additional outcomes, with rationale |  |  | 268-285 |
| **Risk of bias in individual studies** | 14 | Describe anticipated methods for assessing risk of bias of individual studies, including whether this will be done at the outcome or study level, or both; state how this information will be used in data synthesis |  |  | 287-298 |
| ***DATA*** | | | | | |
| **Synthesis** | 15a | Describe criteria under which study data will be quantitatively synthesized |  |  | 300-328 |
|  | 15b | If data are appropriate for quantitative synthesis, describe planned summary measures, methods of handling data, and methods of combining data from studies, including any planned exploration of consistency (e.g., *I* ^2^, Kendall’s tau) |  |  | 302-328 |
|  | 15c | Describe any proposed additional analyses (e.g., sensitivity or subgroup analyses, meta-regression) |  |  |  |
|  | 15d | If quantitative synthesis is not appropriate, describe the type of summary planned |  |  | 301-302 |
| **Meta-bias(es)** | 16 | Specify any planned assessment of meta-bias(es) (e.g., publication bias across studies, selective reporting within studies) |  |  | 330-342 |
| **Confidence in cumulative evidence** | 17 | Describe how the strength of the body of evidence will be assessed (e.g., GRADE) |  |  | 344-361 |

## **PubMed searching history for the fungal diseases searching**

| **Search** | Query | Items found | Time |
| --- | --- | --- | --- |
| **#164** | Search (#161 NOT #163) | 2320 | 02:50:43 |
| **#163** | Search (#162 NOT #156) | 52230 | 02:50:28 |
| **#162** | Search soil[MeSH Terms] | 58163 | 02:50:00 |
| **#161** | Search (#158 NOT #160) | 2320 | 02:49:46 |
| **#160** | Search (#159 NOT #156) | 580777 | 02:49:31 |
| **#159** | Search plant*[MeSH Terms] | 745744 | 02:49:13 |
| **#158** | Search (#154 NOT #157) | 2337 | 02:48:56 |
| **#157** | Search (#155 NOT #156) | 4608200 | 02:48:33 |
| **#156** | Search humans[MeSH Terms] | 17907696 | 02:48:19 |
| **#155** | Search animals[MeSH Terms] | 22515896 | 02:48:09 |
| **#154** | Search (#97 AND #120 AND #153) | 2940 | 02:47:52 |
| **#153** | Search (#121 OR #122 OR #123 OR #124 OR #125 OR #126 OR #127 OR #128 OR #129 OR #130 OR #131 OR #132 OR #133 OR #134 OR #135 OR #136 OR #137 OR #138 OR #139 OR #140 OR #141 OR #142 OR #143 OR #144 OR #145 OR #146 OR #147 OR #148 OR #149 OR #150 OR #151 OR #152) | 462045 | 02:47:23 |
| **#152** | Search laboratory screening[Title/Abstract] | 863 | 02:45:01 |
| **#151** | Search vaccin*[Title/Abstract] | 298956 | 02:44:51 |
| **#150** | Search donor screening[Title/Abstract] | 984 | 02:44:42 |
| **#149** | Search precautionary principle[Title/Abstract] | 750 | 02:44:32 |
| **#148** | Search preventive measures[Title/Abstract] | 19007 | 02:44:21 |
| **#147** | Search blood safety[Title/Abstract] | 1026 | 02:44:11 |
| **#146** | Search serious adverse reactions[Title/Abstract] | 1229 | 02:44:00 |
| **#145** | Search donation material processing[Title/Abstract] | 0 | 02:43:51 |
| **#144** | Search donation material treatment[Title/Abstract] | 0 | 02:43:41 |
| **#143** | Search donor selection criteria[Title/Abstract] | 153 | 02:43:31 |
| **#142** | Search residual risks[Title/Abstract] | 125 | 02:43:21 |
| **#141** | Search donor surveillance[Title/Abstract] | 11 | 02:43:10 |
| **#140** | Search risk mitigation[Title/Abstract] | 962 | 02:43:00 |
| **#139** | Search VCAs don*[Title/Abstract] | 1 | 02:42:49 |
| **#138** | Search vascularized composite allografts don*[Title/Abstract] | 0 | 02:42:38 |
| **#137** | Search intestine don*[Title/Abstract] | 8 | 02:42:11 |
| **#136** | Search lung don*[Title/Abstract] | 373 | 02:42:03 |
| **#135** | Search pancreas don*[Title/Abstract] | 161 | 02:41:52 |
| **#134** | Search kidney don*[Title/Abstract] | 4274 | 02:41:42 |
| **#133** | Search cornea* don*[Title/Abstract] | 532 | 02:41:23 |
| **#132** | Search liver don*[Title/Abstract] | 1698 | 02:41:11 |
| **#131** | Search heart don*[Title/Abstract] | 421 | 02:41:00 |
| **#130** | Search cardiac don*[Title/Abstract] | 123 | 02:40:38 |
| **#129** | Search tissue* donor[Title/Abstract] | 49628 | 02:40:26 |
| **#128** | Search blood components[Title/Abstract] | 5017 | 02:40:16 |
| **#127** | Search blood donor*[Title/Abstract] | 21073 | 02:40:05 |
| **#126** | Search organ* donor[Title/Abstract] | 34015 | 02:39:42 |
| **#125** | Search cell* donor[Title/Abstract] | 64661 | 02:39:32 |
| **#124** | Search human origin donations[Title/Abstract] | 4806 | 02:38:34 |
| **#123** | Search donor-derived[Title/Abstract] | 3178 | 02:38:20 |
| **#122** | Search SoHO[Title/Abstract] | 182 | 02:38:01 |
| **#121** | Search substances of human origin[Title/Abstract] | 66 | 02:37:52 |
| **#120** | Search (#98 OR #99 OR #100 OR #101 OR #102 OR #103 OR #104 OR #105 OR #106 OR #107 OR #108 OR #109 OR #110 OR #111 OR #112 OR #113 OR #114 OR #115 OR #116 OR #117 OR #118 OR #119) | 3501758 | 02:36:40 |
| **#119** | Search blood bank[Title/Abstract] | 3668 | 02:35:19 |
| **#118** | Search allograft recipients[Title/Abstract] | 5144 | 02:35:09 |
| **#117** | Search blood pack[Title/Abstract] | 22 | 02:34:58 |
| **#116** | Search transplant recipients[Title/Abstract] | 42497 | 02:34:48 |
| **#115** | Search autologous transplant[Title/Abstract] | 714 | 02:34:39 |
| **#114** | Search allogeneic transplant[Title/Abstract] | 969 | 02:34:30 |
| **#113** | Search stem cell transplant[Title/Abstract] | 7329 | 02:34:20 |
| **#112** | Search blood transfusion[Title/Abstract] | 38692 | 02:34:10 |
| **#111** | Search transplantation[Title/Abstract] | 340166 | 02:34:01 |
| **#110** | Search surgery[Title/Abstract] | 1162570 | 02:33:51 |
| **#109** | Search harmful substances[Title/Abstract] | 911 | 02:33:41 |
| **#108** | Search infect*[Title/Abstract] | 1673487 | 02:33:25 |
| **#107** | Search contagious[Title/Abstract] | 7544 | 02:33:16 |
| **#106** | Search communicable[Title/Abstract] | 16253 | 02:33:05 |
| **#105** | Search contaminating[Title/Abstract] | 8797 | 02:32:54 |
| **#104** | Search contamination[Title/Abstract] | 108419 | 02:32:45 |
| **#103** | Search transmissible[Title/Abstract] | 12046 | 02:32:33 |
| **#102** | Search transferable[Title/Abstract] | 7054 | 02:32:23 |
| **#101** | Search transmittable[Title/Abstract] | 306 | 02:32:11 |
| **#100** | Search transposal[Title/Abstract] | 8 | 02:32:02 |
| **#99** | Search transmitt*[Title/Abstract] | 136759 | 02:31:42 |
| **#98** | Search transmission[Title/Abstract] | 342882 | 02:31:32 |
| **#97** | Search (#1 OR #2 OR #3 OR #4 OR #5 OR #6 OR #7 OR #8 OR #9 OR #10 OR #11 OR #12 OR #13 OR #14 OR #15 OR #16 OR #17 OR #18 OR #19 OR #20 OR #21 OR #22 OR #23 OR #24 OR #25 OR #26 OR #27 OR #28 OR #29 OR #30 OR #31 OR #32 OR #33 OR #34 OR #35 OR #36 OR #37 OR #38 OR #39 OR #40 OR #41 OR #42 OR #43 OR #44 OR #45 OR #46 OR #47 OR #48 OR #49 OR #50 OR #51 OR #52 OR #53 OR #54 OR #55 OR #56 OR #57 OR #58 OR #59 OR #60 OR #61 OR #62 OR #63 OR #64 OR #65 OR #66 OR #67 OR #68 OR #69 OR #70 OR #71 OR #72 OR #73 OR #74 OR #75 OR #76 OR #77 OR #78 OR #79 OR #80 OR #81 OR #82 OR #83 OR #84 OR #85 OR #86 OR #87 OR #88 OR #89 OR #90 OR #91 OR #92 OR #93 OR #94 OR #95 OR #96) | 358153 | 02:31:06 |
| **#96** | Search Fungal[Title/Abstract] | 102209 | 02:23:52 |
| **#95** | Search Fungi[Title/Abstract] | 77741 | 02:23:41 |
| **#94** | Search Geotrichum[Title/Abstract] | 1074 | 02:23:30 |
| **#93** | Search E. rostratum[Title/Abstract] | 38 | 02:23:19 |
| **#92** | Search Exserohilum rostratum[Title/Abstract] | 92 | 02:23:09 |
| **#91** | Search Scedosporium apiospermum[Title/Abstract] | 537 | 02:22:59 |
| **#90** | Search Trichosporon asahii[Title/Abstract] | 359 | 02:22:49 |
| **#89** | Search Fusarium[Title/Abstract] | 16104 | 02:22:39 |
| **#88** | Search Malassezia furfur[Title/Abstract] | 498 | 02:22:29 |
| **#87** | Search Pseudoallescheria[Title/Abstract] | 28 | 02:22:18 |
| **#86** | Search Non-Fumigatus Aspergilli[Title/Abstract] | 3 | 02:22:08 |
| **#85** | Search Pneumocystis pneumonia[Title/Abstract] | 1537 | 02:21:55 |
| **#84** | Search vaginal yeast infection[Title/Abstract] | 26 | 02:21:45 |
| **#83** | Search Talaromyces marneffei[Title/Abstract] | 110 | 02:21:35 |
| **#82** | Search Talaromycosis[Title/Abstract] | 34 | 02:21:26 |
| **#81** | Search Paracoccidioidomycosis[Title/Abstract] | 1725 | 02:21:16 |
| **#80** | Search S. brasiliensis[Title/Abstract] | 141 | 02:21:07 |
| **#79** | Search Sporotrichosis[Title/Abstract] | 1710 | 02:20:57 |
| **#78** | Search Sporothrix[Title/Abstract] | 1177 | 02:20:48 |
| **#77** | Search P. carinii[Title/Abstract] | 1968 | 02:20:37 |
| **#76** | Search P. jirovecii[Title/Abstract] | 399 | 02:20:26 |
| **#75** | Search Pneumocystis jirovecii[Title/Abstract] | 1244 | 02:20:16 |
| **#74** | Search Eumycetoma[Title/Abstract] | 266 | 02:20:06 |
| **#73** | Search Actinomycetoma[Title/Abstract] | 243 | 02:19:56 |
| **#72** | Search Mycetoma[Title/Abstract] | 1522 | 02:19:45 |
| **#71** | Search S. cerevisiae[Title/Abstract] | 16506 | 02:19:27 |
| **#70** | Search Saccharomyces[Title/Abstract] | 71375 | 02:19:17 |
| **#69** | Search Rhodotorula[Title/Abstract] | 2072 | 02:19:06 |
| **#68** | Search Absidia[Title/Abstract] | 506 | 02:18:52 |
| **#67** | Search Lichtheimia[Title/Abstract] | 152 | 02:18:43 |
| **#66** | Search Apophysomyces[Title/Abstract] | 135 | 02:18:31 |
| **#65** | Search Cunninghamella bertholletiae[Title/Abstract] | 107 | 02:18:21 |
| **#64** | Search Syncephalastrum[Title/Abstract] | 155 | 02:18:12 |
| **#63** | Search Rhizomucor[Title/Abstract] | 586 | 02:18:01 |
| **#62** | Search Mucor[Title/Abstract] | 2718 | 02:17:51 |
| **#61** | Search Rhizopus[Title/Abstract] | 3340 | 02:17:41 |
| **#60** | Search Mucormycosis[Title/Abstract] | 3341 | 02:17:31 |
| **#59** | Search Histoplasmosis[Title/Abstract] | 5657 | 02:17:22 |
| **#58** | Search valley fever[Title/Abstract] | 1941 | 02:17:10 |
| **#57** | Search coccidioidomycosis[Title/Abstract] | 2531 | 02:17:00 |
| **#56** | Search Coccidioides[Title/Abstract] | 1605 | 02:16:50 |
| **#55** | Search Blastomycosis[Title/Abstract] | 2364 | 02:16:39 |
| **#54** | Search A. nidulans[Title/Abstract] | 1855 | 02:16:29 |
| **#53** | Search A. calidoustus[Title/Abstract] | 27 | 02:16:19 |
| **#52** | Search A. versicolor[Title/Abstract] | 248 | 02:16:08 |
| **#51** | Search A. terreus[Title/Abstract] | 685 | 02:15:58 |
| **#50** | Search A. niger[Title/Abstract] | 2985 | 02:15:48 |
| **#49** | Search A. flavus[Title/Abstract] | 2272 | 02:15:38 |
| **#48** | Search Aspergillosis[Title/Abstract] | 13023 | 02:15:28 |
| **#47** | Search A. fumigatus[Title/Abstract] | 4280 | 02:15:18 |
| **#46** | Search Aspergillus[Title/Abstract] | 41954 | 02:15:08 |
| **#45** | Search C. neoformans var. grubii[Title/Abstract] | 170 | 02:14:57 |
| **#44** | Search C. gattii[Title/Abstract] | 575 | 02:14:46 |
| **#43** | Search C. neoformans[Title/Abstract] | 3285 | 02:14:37 |
| **#42** | Search Cryptococcus[Title/Abstract] | 10378 | 02:14:26 |
| **#41** | Search Basidiomycota[Title/Abstract] | 1372 | 02:14:15 |
| **#40** | Search C. rugosa[Title/Abstract] | 190 | 02:14:04 |
| **#39** | Search C. kefyr[Title/Abstract] | 262 | 02:13:53 |
| **#38** | Search C. dubliniensis[Title/Abstract] | 712 | 02:13:44 |
| **#37** | Search C. auris[Title/Abstract] | 192 | 02:13:34 |
| **#36** | Search candidemia[Title/Abstract] | 2289 | 02:13:24 |
| **#35** | Search Candidiasis[Title/Abstract] | 14986 | 02:13:13 |
| **#34** | Search C. guilliermondii[Title/Abstract] | 577 | 02:13:03 |
| **#33** | Search C. krusei[Title/Abstract] | 1655 | 02:12:52 |
| **#32** | Search C. lusitaniae[Title/Abstract] | 386 | 02:12:41 |
| **#31** | Search C. tropicalis[Title/Abstract] | 2648 | 02:12:28 |
| **#30** | Search C. parapsilosis[Title/Abstract] | 2365 | 02:12:17 |
| **#29** | Search C. glabrata[Title/Abstract] | 3028 | 02:12:07 |
| **#28** | Search C. albicans[Title/Abstract] | 15893 | 02:11:57 |
| **#27** | Search Candida[Title/Abstract] | 58213 | 02:11:47 |
| **#26** | Search Epidermophyton[Title/Abstract] | 801 | 02:11:38 |
| **#25** | Search Microsporum[Title/Abstract] | 2847 | 02:11:28 |
| **#24** | Search Trichophyton[Title/Abstract] | 5813 | 02:11:17 |
| **#23** | Search Lacazia[Title/Abstract] | 71 | 02:11:06 |
| **#22** | Search farciminosum[Title/Abstract] | 39 | 02:10:57 |
| **#21** | Search capsulatum[Title/Abstract] | 2924 | 02:10:47 |
| **#20** | Search Penicillium marneffei[Title/Abstract] | 604 | 02:10:36 |
| **#19** | Search Histoplasma[Title/Abstract] | 3376 | 02:10:23 |
| **#18** | Search Blastomyces[Title/Abstract] | 1185 | 02:10:12 |
| **#17** | Search C. posadasii[Title/Abstract] | 113 | 02:10:01 |
| **#16** | Search C. immitis[Title/Abstract] | 376 | 02:09:50 |
| **#15** | Search P. lutzii[Title/Abstract] | 79 | 02:09:40 |
| **#14** | Search P. brasiliensis[Title/Abstract] | 957 | 02:09:30 |
| **#13** | Search Paracoccidioides[Title/Abstract] | 1747 | 02:09:20 |
| **#12** | Search Ajellomyces capsulatus[Title/Abstract] | 12 | 02:09:09 |
| **#11** | Search Ajellomyces dermatitidis[Title/Abstract] | 22 | 02:08:59 |
| **#10** | Search B. dermatitidis[Title/Abstract] | 333 | 02:08:47 |
| **#9** | Search Ascomycota[Title/Abstract] | 1930 | 02:08:37 |
| **#8** | Search Basidiobolus ranarum[Title/Abstract] | 110 | 02:08:26 |
| **#7** | Search C. incongruus[Title/Abstract] | 6 | 02:08:15 |
| **#6** | Search C. coronatus[Title/Abstract] | 54 | 02:08:05 |
| **#5** | Search Conidiobolus[Title/Abstract] | 198 | 02:07:55 |
| **#4** | Search entomophthoromycota[Title/Abstract] | 24 | 02:07:44 |
| **#3** | Search yeasts[Title/Abstract] | 23061 | 02:07:33 |
| **#2** | Search mould[Title/Abstract] | 4342 | 02:07:23 |
| **#1** | Search mold[Title/Abstract] | 11394 | 02:07:09 |

## **PubMed searching history for the parasitic diseases searching**

| **Search** | Query | Items found | Time |
| --- | --- | --- | --- |
| **#139** | Search (#136 NOT #138) | 1895 | 09:56:27 |
| **#138** | Search (#137 NOT #131) | 52230 | 09:56:12 |
| **#137** | Search soil[MeSH Terms] | 58163 | 09:55:53 |
| **#136** | Search (#133 NOT #135) | 1895 | 09:55:41 |
| **#135** | Search (#134 NOT #131) | 580777 | 09:55:25 |
| **#134** | Search plant*[MeSH Terms] | 745744 | 09:55:10 |
| **#133** | Search (#129 NOT #132) | 1898 | 09:54:57 |
| **#132** | Search (#130 NOT #131) | 4608200 | 09:54:46 |
| **#131** | Search humans[MeSH Terms] | 17907696 | 09:54:33 |
| **#130** | Search animals[MeSH Terms] | 22515896 | 09:54:22 |
| **#129** | Search (#73 AND #96 AND #128) | 2081 | 09:53:48 |
| **#128** | Search (#97 OR #98 OR #99 OR #100 OR #101 OR #102 OR #103 OR #104 OR #105 OR #106 OR #107 OR #108 OR #109 OR #110 OR #111 OR #112 OR #113 OR #114 OR #115 OR #116 OR #117 OR #118 OR #119 OR #120 OR #121 OR #122 OR #123 OR #124 OR #125 OR #126 OR #127) | 166270 | 09:53:23 |
| **#127** | Search laboratory screening[Title/Abstract] | 863 | 09:51:25 |
| **#126** | Search donor screening[Title/Abstract] | 985 | 09:46:15 |
| **#125** | Search precautionary principle[Title/Abstract] | 750 | 09:46:06 |
| **#124** | Search preventive measures[Title/Abstract] | 19008 | 09:45:56 |
| **#123** | Search blood safety[Title/Abstract] | 1026 | 09:45:47 |
| **#122** | Search serious adverse reactions[Title/Abstract] | 1229 | 09:45:35 |
| **#121** | Search donation material processing[Title/Abstract] | 0 | 09:45:26 |
| **#120** | Search donation material treatment[Title/Abstract] | 0 | 09:45:16 |
| **#119** | Search donor selection criteria[Title/Abstract] | 153 | 09:45:06 |
| **#118** | Search residual risks[Title/Abstract] | 125 | 09:44:56 |
| **#117** | Search donor surveillance[Title/Abstract] | 11 | 09:44:46 |
| **#116** | Search risk mitigation[Title/Abstract] | 962 | 09:44:36 |
| **#115** | Search VCAs don*[Title/Abstract] | 1 | 09:44:27 |
| **#114** | Search vascularized composite allografts don*[Title/Abstract] | 0 | 09:43:54 |
| **#113** | Search intestine don*[Title/Abstract] | 8 | 09:43:45 |
| **#112** | Search lung don*[Title/Abstract] | 373 | 09:43:35 |
| **#111** | Search pancreas don*[Title/Abstract] | 161 | 09:43:25 |
| **#110** | Search kidney don*[Title/Abstract] | 4278 | 09:43:15 |
| **#109** | Search cornea* don*[Title/Abstract] | 533 | 09:43:05 |
| **#108** | Search liver don*[Title/Abstract] | 1699 | 09:42:56 |
| **#107** | Search heart don*[Title/Abstract] | 421 | 09:42:47 |
| **#106** | Search cardiac don*[Title/Abstract] | 123 | 09:42:38 |
| **#105** | Search tissue* donor[Title/Abstract] | 49631 | 09:42:28 |
| **#104** | Search blood components[Title/Abstract] | 5017 | 09:42:17 |
| **#103** | Search blood donor*[Title/Abstract] | 21074 | 09:42:06 |
| **#102** | Search organ* donor[Title/Abstract] | 34023 | 09:41:56 |
| **#101** | Search cell* donor[Title/Abstract] | 64670 | 09:41:46 |
| **#100** | Search human origin donations[Title/Abstract] | 4806 | 09:41:36 |
| **#99** | Search donor-derived[Title/Abstract] | 3180 | 09:41:27 |
| **#98** | Search SoHO[Title/Abstract] | 182 | 09:41:17 |
| **#97** | Search substances of human origin[Title/Abstract] | 66 | 09:41:07 |
| **#96** | Search (#74 OR #75 OR #76 OR #77 OR #78 OR #79 OR #80 OR #81 OR #82 OR #83 OR #84 OR #85 OR #86 OR #87 OR #88 OR #89 OR #90 OR #91 OR #92 OR #93 OR #94 OR #95) | 3502091 | 09:40:24 |
| **#95** | Search blood bank[Title/Abstract] | 3668 | 09:38:33 |
| **#94** | Search allograft recipients[Title/Abstract] | 5144 | 09:38:23 |
| **#93** | Search blood pack[Title/Abstract] | 22 | 09:38:13 |
| **#92** | Search transplant recipients[Title/Abstract] | 42509 | 09:38:03 |
| **#91** | Search autologous transplant[Title/Abstract] | 714 | 09:37:54 |
| **#90** | Search allogeneic transplant[Title/Abstract] | 969 | 09:37:44 |
| **#89** | Search stem cell transplant[Title/Abstract] | 7329 | 09:37:33 |
| **#88** | Search blood transfusion[Title/Abstract] | 38693 | 09:37:24 |
| **#87** | Search transplantation[Title/Abstract] | 340223 | 09:37:14 |
| **#86** | Search surgery[Title/Abstract] | 1162674 | 09:37:01 |
| **#85** | Search harmful substances[Title/Abstract] | 911 | 09:36:50 |
| **#84** | Search infect*[Title/Abstract] | 1673639 | 09:36:41 |
| **#83** | Search contagious[Title/Abstract] | 7545 | 09:36:31 |
| **#82** | Search communicable[Title/Abstract] | 16255 | 09:36:21 |
| **#81** | Search contaminating[Title/Abstract] | 8798 | 09:36:11 |
| **#80** | Search contamination[Title/Abstract] | 108430 | 09:36:00 |
| **#79** | Search transmissible[Title/Abstract] | 12046 | 09:35:50 |
| **#78** | Search transferable[Title/Abstract] | 7055 | 09:35:40 |
| **#77** | Search transmittable[Title/Abstract] | 306 | 09:35:31 |
| **#76** | Search transposal[Title/Abstract] | 8 | 09:35:21 |
| **#75** | Search transmitt*[Title/Abstract] | 136773 | 09:33:35 |
| **#74** | Search transmission[Title/Abstract] | 342913 | 09:33:25 |
| **#73** | Search (#1 OR #2 OR #3 OR #4 OR #5 OR #6 OR #7 OR #8 OR #9 OR #10 OR #11 OR #12 OR #13 OR #14 OR #15 OR #16 OR #17 OR #18 OR #19 OR #20 OR #21 OR #22 OR #23 OR #24 OR #25 OR #26 OR #27 OR #28 OR #29 OR #30 OR #31 OR #32 OR #33 OR #34 OR #35 OR #36 OR #37 OR #38 OR #39 OR #40 OR #41 OR #42 OR #43 OR #44 OR #45 OR #46 OR #47 OR #48 OR #49 OR #50 OR #51 OR #52 OR #53 OR #54 OR #55 OR #56 OR #57 OR #58 OR #59 OR #60 OR #61 OR #62 OR #63 OR #64 OR #65 OR #66 OR #67 OR #68 OR #69 OR #70 OR #71 OR #72) | 311138 | 09:32:53 |
| **#72** | Search P. knowlesi[Title/Abstract] | 659 | 09:26:44 |
| **#71** | Search P. ovale[Title/Abstract] | 742 | 09:26:34 |
| **#70** | Search P. malariae[Title/Abstract] | 805 | 09:26:24 |
| **#69** | Search P. vivax[Title/Abstract] | 4667 | 09:26:13 |
| **#68** | Search P. falciparum[Title/Abstract] | 15144 | 09:26:02 |
| **#67** | Search Trichomonas vaginalis[Title/Abstract] | 4554 | 09:25:52 |
| **#66** | Search Trichomoniasis[Title/Abstract] | 3312 | 09:25:41 |
| **#65** | Search T. gondii[Title/Abstract] | 7978 | 09:25:31 |
| **#64** | Search Toxoplasmosis[Title/Abstract] | 15118 | 09:25:21 |
| **#63** | Search Toxoplasma[Title/Abstract] | 17774 | 09:25:11 |
| **#62** | Search S. stercoralis[Title/Abstract] | 784 | 09:25:01 |
| **#61** | Search Strongyloides[Title/Abstract] | 3830 | 09:22:08 |
| **#60** | Search Strongyloidiasis[Title/Abstract] | 2024 | 09:21:58 |
| **#59** | Search P. carinii[Title/Abstract] | 1968 | 09:21:48 |
| **#58** | Search P. jirovecii[Title/Abstract] | 399 | 09:21:38 |
| **#57** | Search Pneumocystis[Title/Abstract] | 10721 | 09:21:28 |
| **#56** | Search enterobiasis[Title/Abstract] | 501 | 09:21:18 |
| **#55** | Search hominis[Title/Abstract] | 6198 | 09:21:08 |
| **#54** | Search Sarcoptes scabiei[Title/Abstract] | 788 | 09:20:58 |
| **#53** | Search Scabies[Title/Abstract] | 3450 | 09:20:46 |
| **#52** | Search Vittaforma corneae[Title/Abstract] | 45 | 09:20:35 |
| **#51** | Search Tubulinosema acridophagus[Title/Abstract] | 5 | 09:20:18 |
| **#50** | Search Trachipleistophora[Title/Abstract] | 40 | 09:20:07 |
| **#49** | Search Pleistophora ronneafiei[Title/Abstract] | 2 | 09:19:56 |
| **#48** | Search Nosema ocularum[Title/Abstract] | 0 | 09:19:45 |
| **#47** | Search Microsporidium[Title/Abstract] | 501 | 09:19:35 |
| **#46** | Search Encephalitozoon[Title/Abstract] | 1068 | 09:19:25 |
| **#45** | Search Anncaliia[Title/Abstract] | 26 | 09:19:15 |
| **#44** | Search Microsporidiosis[Title/Abstract] | 697 | 09:19:04 |
| **#43** | Search Plasmodium[Title/Abstract] | 47574 | 09:18:54 |
| **#42** | Search Anopheles[Title/Abstract] | 13148 | 09:18:44 |
| **#41** | Search Malaria[Title/Abstract] | 77867 | 09:18:27 |
| **#40** | Search Leishmaniasis[Title/Abstract] | 21360 | 09:18:16 |
| **#39** | Search Leishmania[Title/Abstract] | 23385 | 09:18:05 |
| **#38** | Search Giardiasis[Title/Abstract] | 3113 | 09:17:54 |
| **#37** | Search G. duodenalis[Title/Abstract] | 706 | 09:17:43 |
| **#36** | Search G. lamblia[Title/Abstract] | 1163 | 09:17:32 |
| **#35** | Search G. intestinalis[Title/Abstract] | 468 | 09:15:30 |
| **#34** | Search Giardia[Title/Abstract] | 7796 | 09:14:23 |
| **#33** | Search Enterobius vermicularis[Title/Abstract] | 1035 | 09:14:11 |
| **#32** | Search E. vermicularis[Title/Abstract] | 275 | 09:13:59 |
| **#31** | Search Pinworm[Title/Abstract] | 469 | 09:13:48 |
| **#30** | Search Isospora belli[Title/Abstract] | 389 | 09:13:38 |
| **#29** | Search C. belli[Title/Abstract] | 27 | 09:13:27 |
| **#28** | Search Cystoisospora belli[Title/Abstract] | 55 | 09:13:13 |
| **#27** | Search Cystoisosporiasis[Title/Abstract] | 18 | 09:12:58 |
| **#26** | Search pubic “crab”[Title/Abstract] | 15 | 09:12:46 |
| **#25** | Search Pubic lice[Title/Abstract] | 56 | 09:12:34 |
| **#24** | Search Chagas disease[Title/Abstract] | 11176 | 09:12:23 |
| **#23** | Search Capillaria hepatica[Title/Abstract] | 220 | 09:12:11 |
| **#22** | Search capillariasis[Title/Abstract] | 169 | 09:11:57 |
| **#21** | Search C. hepatica[Title/Abstract] | 65 | 09:11:41 |
| **#20** | Search B. mandrillaris[Title/Abstract] | 102 | 09:11:30 |
| **#19** | Search Balamuthia[Title/Abstract] | 267 | 09:11:18 |
| **#18** | Search B. duncani[Title/Abstract] | 14 | 09:11:07 |
| **#17** | Search B. divergens[Title/Abstract] | 257 | 09:10:51 |
| **#16** | Search Babesiosis[Title/Abstract] | 2356 | 09:10:38 |
| **#15** | Search B. microti[Title/Abstract] | 570 | 09:10:27 |
| **#14** | Search Babesia[Title/Abstract] | 4978 | 09:10:16 |
| **#13** | Search T. cruzi[Title/Abstract] | 8219 | 09:10:03 |
| **#12** | Search Trypanosoma[Title/Abstract] | 26439 | 09:09:53 |
| **#11** | Search Amebiasis[Title/Abstract] | 4786 | 09:09:41 |
| **#10** | Search E. dispar[Title/Abstract] | 506 | 09:09:28 |
| **#9** | Search E. histolytica[Title/Abstract] | 2850 | 09:09:18 |
| **#8** | Search Entamoeba[Title/Abstract] | 7328 | 09:09:08 |
| **#7** | Search T. b. rhodesiense[Title/Abstract] | 414 | 09:08:57 |
| **#6** | Search T. b. gambiense[Title/Abstract] | 419 | 09:08:46 |
| **#5** | Search T. brucei[Title/Abstract] | 3247 | 09:08:35 |
| **#4** | Search Trypanosomiasis[Title/Abstract] | 6731 | 09:08:25 |
| **#3** | Search parasitism[Title/Abstract] | 8723 | 09:08:13 |
| **#2** | Search parasitic[Title/Abstract] | 45219 | 09:08:03 |
| **#1** | Search parasites[Title/Abstract] | 70837 | 09:07:52 |

## **EMBASE searching history for the fungal diseases searching**

| **Searches** | **Results** | **Type** |
| --- | --- | --- |
| 1 | mold.ab,ti. | 11704 |
| 2 | mould.ab,ti. | 5448 |
| 3 | yeasts.ab,ti. | 22266 |
| 4 | entomophthoromycota.ab,ti. | 15 |
| 5 | Conidiobolus.ab,ti. | 219 |
| 6 | "C. coronatus".ab,ti. | 51 |
| 7 | "C. incongruus".ab,ti. | 6 |
| 8 | Basidiobolus ranarum.ab,ti. | 103 |
| 9 | Ascomycota.ab,ti. | 1704 |
| 10 | "B. dermatitidis".ab,ti. | 321 |
| 11 | Ajellomyces dermatitidis.ab,ti. | 16 |
| 12 | Ajellomyces capsulatus.ab,ti. | 13 |
| 13 | Paracoccidioides.ab,ti. | 1869 |
| 14 | "P. brasiliensis".ab,ti. | 1043 |
| 15 | "P. lutzii".ab,ti. | 100 |
| 16 | "C. immitis".ab,ti. | 349 |
| 17 | "C. posadasii".ab,ti. | 131 |
| 18 | Blastomyces.ab,ti. | 1068 |
| 19 | Histoplasma.ab,ti. | 3334 |
| 20 | Penicillium marneffei.ab,ti. | 700 |
| 21 | capsulatum.ab,ti. | 2723 |
| 22 | farciminosum.ab,ti. | 32 |
| 23 | Lacazia.ab,ti. | 84 |
| 24 | Trichophyton.ab,ti. | 6164 |
| 25 | Microsporum.ab,ti. | 2964 |
| 26 | Epidermophyton.ab,ti. | 900 |
| 27 | Candida.ab,ti. | 70504 |
| 28 | "C. albicans".ab,ti. | 19291 |
| 29 | "C. glabrata".ab,ti. | 4280 |
| 30 | "C. parapsilosis".ab,ti. | 3334 |
| 31 | "C. tropicalis".ab,ti. | 3468 |
| 32 | "C. lusitaniae".ab,ti. | 540 |
| 33 | "C. krusei".ab,ti. | 2353 |
| 34 | "C. guilliermondii".ab,ti. | 733 |
| 35 | Candidiasis.ab,ti. | 17859 |
| 36 | candidemia.ab,ti. | 3207 |
| 37 | "C. auris".ab,ti. | 296 |
| 38 | "C. dubliniensis".ab,ti. | 930 |
| 39 | "C. kefyr".ab,ti. | 407 |
| 40 | "C. rugosa".ab,ti. | 270 |
| 41 | Basidiomycota.ab,ti. | 1226 |
| 42 | Cryptococcus.ab,ti. | 11515 |
| 43 | "C. neoformans".ab,ti. | 3770 |
| 44 | "C. gattii".ab,ti. | 762 |
| 45 | "C. neoformans var. grubii".ab,ti. | 222 |
| 46 | Aspergillus.ab,ti. | 50623 |
| 47 | "A. fumigatus".ab,ti. | 5494 |
| 48 | Aspergillosis.ab,ti. | 16106 |
| 49 | "A. flavus".ab,ti. | 2694 |
| 50 | "A. niger".ab,ti. | 3867 |
| 51 | "A. terreus".ab,ti. | 914 |
| 52 | "A. versicolor".ab,ti. | 300 |
| 53 | "A. calidoustus".ab,ti. | 35 |
| 54 | "A. nidulans".ab,ti. | 1889 |
| 55 | Blastomycosis.ab,ti. | 1496 |
| 56 | "Coccidioides[".ab,ti. | 1538 |
| 57 | coccidioidomycosis.ab,ti. | 2062 |
| 58 | valley fever.ab,ti. | 1974 |
| 59 | Histoplasmosis.ab,ti. | 4804 |
| 60 | Mucormycosis.ab,ti. | 3967 |
| 61 | Rhizopus.ab,ti. | 4266 |
| 62 | Mucor.ab,ti. | 3338 |
| 63 | Rhizomucor.ab,ti. | 853 |
| 64 | Syncephalastrum.ab,ti. | 176 |
| 65 | Cunninghamella.ab,ti. | 803 |
| 66 | Apophysomyces.ab,ti. | 150 |
| 67 | Lichtheimia.ab,ti. | 235 |
| 68 | Absidia.ab,ti. | 592 |
| 69 | Rhodotorula.ab,ti. | 2141 |
| 70 | Saccharomyces.ab,ti. | 72366 |
| 71 | "S. cerevisiae".ab,ti. | 17424 |
| 72 | Mycetoma.ab,ti. | 1377 |
| 73 | Actinomycetoma.ab,ti. | 299 |
| 74 | Eumycetoma.ab,ti. | 343 |
| 75 | Pneumocystis jirovecii.ab,ti. | 1916 |
| 76 | "P. jirovecii".ab,ti. | 569 |
| 77 | "P. carinii".ab,ti. | 2010 |
| 78 | Sporothrix.ab,ti. | 1315 |
| 79 | Sporotrichosis.ab,ti. | 1545 |
| 80 | "S. brasiliensis".ab,ti. | 188 |
| 81 | Paracoccidioidomycosis.ab,ti. | 1892 |
| 82 | Talaromycosis.ab,ti. | 45 |
| 83 | Talaromyces marneffei.ab,ti. | 135 |
| 84 | vaginal yeast.ab,ti. | 164 |
| 85 | Pneumocystis pneumonia.ab,ti. | 2079 |
| 86 | Non-Fumigatus Aspergilli.ab,ti. | 3 |
| 87 | Pseudoallescheria.ab,ti. | 43 |
| 88 | Malassezia furfur.ab,ti. | 673 |
| 89 | Fusarium.ab,ti. | 17202 |
| 90 | Trichosporon asahii.ab,ti. | 453 |
| 91 | Scedosporium apiospermum.ab,ti. | 705 |
| 92 | Exserohilum rostratum.ab,ti. | 106 |
| 93 | "E. rostratum".ab,ti. | 38 |
| 94 | Geotrichum.ab,ti. | 1214 |
| 95 | Fungi.ab,ti. | 82422 |
| 96 | Fungal.ab,ti. | 122626 |
| 97 | 1 or 2 or 3 or 4 or 5 or 6 or 7 or 8 or 9 or 10 or 11 or 12 or 13 or 14 or 15 or 16 or 17 or 18 or 19 or 20 or 21 or 22 or 23 or 24 or 25 or 26 or 27 or 28 or 29 or 30 or 31 or 32 or 33 or 34 or 35 or 36 or 37 or 38 or 39 or 40 or 41 or 42 or 43 or 44 or 45 or 46 or 47 or 48 or 49 or 50 or 51 or 52 or 53 or 54 or 55 or 56 or 57 or 58 or 59 or 60 or 61 or 62 or 63 or 64 or 65 or 66 or 67 or 68 or 69 or 70 or 71 or 72 or 73 or 74 or 75 or 76 or 77 or 78 or 79 or 80 or 81 or 82 or 83 or 84 or 85 or 86 or 87 or 88 or 89 or 90 or 91 or 92 or 93 or 94 or 95 or 96 | 388219 |
| 98 | transmission.ab,ti. | 366379 |
| 99 | "transmitt*".ab,ti. | 148968 |
| 100 | transposal.ab,ti. | 9 |
| 101 | transmittable.ab,ti. | 416 |
| 102 | transferable.ab,ti. | 7618 |
| 103 | transmissible.ab,ti. | 12894 |
| 104 | contamination.ab,ti. | 124327 |
| 105 | contaminating.ab,ti. | 9329 |
| 106 | communicable.ab,ti. | 13473 |
| 107 | contagious.ab,ti. | 8043 |
| 108 | "infect*".ab,ti. | 1993706 |
| 109 | harmful substances.ab,ti. | 1129 |
| 110 | surgery.ab,ti. | 1463893 |
| 111 | transplantation.ab,ti. | 473291 |
| 112 | blood transfusion.ab,ti. | 48805 |
| 113 | stem cell transplant.ab,ti. | 16811 |
| 114 | allogeneic transplant.ab,ti. | 2238 |
| 115 | autologous transplant.ab,ti. | 1578 |
| 116 | transplant recipients.ab,ti. | 67605 |
| 117 | blood pack.ab,ti. | 41 |
| 118 | allograft recipients.ab,ti. | 6875 |
| 119 | blood bank.ab,ti. | 7176 |
| 120 | 98 or 99 or 100 or 101 or 102 or 103 or 104 or 105 or 106 or 107 or 108 or 109 or 110 or 111 or 112 or 113 or 114 or 115 or 116 or 117 or 118 or 119 | 4218031 |
| 121 | substances of human origin.ab,ti. | 27 |
| 122 | SoHO.ab,ti. | 219 |
| 123 | donor-derived.ab,ti. | 5216 |
| 124 | human origin donations.ab,ti. | 0 |
| 125 | "cell* donor".ab,ti. | 1540 |
| 126 | "organ* donor".ab,ti. | 3107 |
| 127 | "blood donor*".ab,ti. | 29218 |
| 128 | blood components.ab,ti. | 8452 |
| 129 | "tissue* donor".ab,ti. | 312 |
| 130 | "cardiac don*".ab,ti. | 240 |
| 131 | "heart don*".ab,ti. | 745 |
| 132 | "liver don*".ab,ti. | 3102 |
| 133 | "cornea* don*".ab,ti. | 590 |
| 134 | "kidney don*".ab,ti. | 7418 |
| 135 | "pancreas don*".ab,ti. | 303 |
| 136 | "lung don*".ab,ti. | 708 |
| 137 | "intestine don*".ab,ti. | 19 |
| 138 | "vascularized composite allografts don*".ab,ti. | 0 |
| 139 | "VCAs don*".ab,ti. | 0 |
| 140 | risk mitigation.ab,ti. | 1335 |
| 141 | donor surveillance.ab,ti. | 24 |
| 142 | residual risks.ab,ti. | 212 |
| 143 | donor selection criteria.ab,ti. | 340 |
| 144 | donation material treatment.ab,ti. | 0 |
| 145 | donation material processing.ab,ti. | 0 |
| 146 | serious adverse reactions.ab,ti. | 1922 |
| 147 | blood safety.ab,ti. | 1976 |
| 148 | preventive measures.ab,ti. | 24447 |
| 149 | precautionary principle.ab,ti. | 914 |
| 150 | donor screening.ab,ti. | 1727 |
| 151 | "vaccin*".ab,ti. | 327980 |
| 152 | laboratory screening.ab,ti. | 1266 |
| 153 | 121 or 122 or 123 or 124 or 125 or 126 or 127 or 128 or 129 or 130 or 131 or 132 or 133 or 134 or 135 or 136 or 137 or 138 or 139 or 140 or 141 or 142 or 143 or 144 or 145 or 146 or 147 or 148 or 149 or 150 or 151 or 152 | 415561 |
| 154 | 97 and 120 and 153 | 2853 |
| 155 | limit 154 to human | 1847 |

##

## **EMBASE searching history for the parasitic diseases searching**

| **Searches** | **Results** | **Type** |
| --- | --- | --- |
| 1 | parasites.ab,ti. | 75658 |
| 2 | parasitic.ab,ti. | 44690 |
| 3 | parasitism.ab,ti. | 7447 |
| 4 | Trypanosomiasis.ab,ti. | 4808 |
| 5 | "T. brucei".ab,ti. | 3235 |
| 6 | "T. b. gambiense".ab,ti. | 331 |
| 7 | "T. b. rhodesiense".ab,ti. | 358 |
| 8 | Entamoeba.ab,ti. | 7153 |
| 9 | "E. histolytica".ab,ti. | 2846 |
| 10 | "E. dispar".ab,ti. | 573 |
| 11 | Amebiasis.ab,ti. | 1734 |
| 12 | Trypanosoma.ab,ti. | 25521 |
| 13 | "T. cruzi".ab,ti. | 8940 |
| 14 | Babesia.ab,ti. | 4929 |
| 15 | "B. microti".ab,ti. | 668 |
| 16 | Babesiosis.ab,ti. | 2481 |
| 17 | "B. divergens".ab,ti. | 269 |
| 18 | "B. duncani".ab,ti. | 23 |
| 19 | Balamuthia.ab,ti. | 299 |
| 20 | "B. mandrillaris".ab,ti. | 112 |
| 21 | "C. hepatica".ab,ti. | 68 |
| 22 | capillariasis.ab,ti. | 151 |
| 23 | Capillaria hepatica.ab,ti. | 162 |
| 24 | Chagas disease.ab,ti. | 11947 |
| 25 | pubic crab.ab,ti. | 4 |
| 26 | Cystoisosporiasis.ab,ti. | 17 |
| 27 | Cystoisospora belli.ab,ti. | 63 |
| 28 | "C. belli".ab,ti. | 28 |
| 29 | Isospora belli.ab,ti. | 399 |
| 30 | Pinworm.ab,ti. | 435 |
| 31 | "E. vermicularis".ab,ti. | 295 |
| 32 | Enterobius vermicularis.ab,ti. | 1054 |
| 33 | Giardia.ab,ti. | 8962 |
| 34 | "G. intestinalis".ab,ti. | 510 |
| 35 | "G. lamblia".ab,ti. | 1280 |
| 36 | "G. duodenalis".ab,ti. | 774 |
| 37 | Giardiasis.ab,ti. | 2878 |
| 38 | Leishmania.ab,ti. | 25544 |
| 39 | Leishmaniasis.ab,ti. | 22430 |
| 40 | Malaria.ab,ti. | 84917 |
| 41 | Pubic lice.ab,ti. | 69 |
| 42 | Anopheles.ab,ti. | 13250 |
| 43 | Plasmodium.ab,ti. | 51817 |
| 44 | Microsporidiosis.ab,ti. | 717 |
| 45 | Anncaliia.ab,ti. | 30 |
| 46 | Encephalitozoon.ab,ti. | 1050 |
| 47 | Microsporidium.ab,ti. | 509 |
| 48 | Nosema ocularum.ab,ti. | 0 |
| 49 | Pleistophora ronneafiei.ab,ti. | 1 |
| 50 | Trachipleistophora.ab,ti. | 43 |
| 51 | Tubulinosema acridophagus.ab,ti. | 5 |
| 52 | Vittaforma corneae.ab,ti. | 46 |
| 53 | Scabies.ab,ti. | 3691 |
| 54 | Sarcoptes scabiei.ab,ti. | 883 |
| 55 | hominis.ab,ti. | 6205 |
| 56 | enterobiasis.ab,ti. | 376 |
| 57 | Pneumocystis.ab,ti. | 12905 |
| 58 | "P. jirovecii".ab,ti. | 569 |
| 59 | "P. carinii".ab,ti. | 2010 |
| 60 | Strongyloidiasis.ab,ti. | 1982 |
| 61 | Strongyloides.ab,ti. | 4252 |
| 62 | "S. stercoralis".ab,ti. | 933 |
| 63 | Toxoplasma.ab,ti. | 18331 |
| 64 | Toxoplasmosis.ab,ti. | 12918 |
| 65 | "T. gondii".ab,ti. | 8643 |
| 66 | Trichomoniasis.ab,ti. | 2485 |
| 67 | Trichomonas vaginalis.ab,ti. | 4602 |
| 68 | "P. falciparum".ab,ti. | 18050 |
| 69 | "P. vivax".ab,ti. | 5738 |
| 70 | "P. malariae".ab,ti. | 980 |
| 71 | "P. ovale".ab,ti. | 911 |
| 72 | "P. knowlesi".ab,ti. | 687 |
| 73 | 1 or 2 or 3 or 4 or 5 or 6 or 7 or 8 or 9 or 10 or 11 or 12 or 13 or 14 or 15 or 16 or 17 or 18 or 19 or 20 or 21 or 22 or 23 or 24 or 25 or 26 or 27 or 28 or 29 or 30 or 31 or 32 or 33 or 34 or 35 or 36 or 37 or 38 or 39 or 40 or 41 or 42 or 43 or 44 or 45 or 46 or 47 or 48 or 49 or 50 or 51 or 52 or 53 or 54 or 55 or 56 or 57 or 58 or 59 or 60 or 61 or 62 or 63 or 64 or 65 or 66 or 67 or 68 or 69 or 70 or 71 or 72 | 310513 |
| 74 | transmission.ab,ti. | 366379 |
| 75 | "transmitt*".ab,ti. | 148968 |
| 76 | transposal.ab,ti. | 9 |
| 77 | transmittable.ab,ti. | 416 |
| 78 | transferable.ab,ti. | 7618 |
| 79 | transmissible.ab,ti. | 12894 |
| 80 | contamination.ab,ti. | 124327 |
| 81 | contaminating.ab,ti. | 9329 |
| 82 | communicable.ab,ti. | 13473 |
| 83 | contagious.ab,ti. | 8043 |
| 84 | "infect*".ab,ti. | 1993706 |
| 85 | harmful substances.ab,ti. | 1129 |
| 86 | surgery.ab,ti. | 1463893 |
| 87 | transplantation.ab,ti. | 473291 |
| 88 | blood transfusion.ab,ti. | 48805 |
| 89 | stem cell transplant.ab,ti. | 16811 |
| 90 | allogeneic transplant.ab,ti. | 2238 |
| 91 | autologous transplant.ab,ti. | 1578 |
| 92 | transplant recipients.ab,ti. | 67605 |
| 93 | blood pack.ab,ti. | 41 |
| 94 | allograft recipients.ab,ti. | 6875 |
| 95 | blood bank.ab,ti. | 7176 |
| 96 | 74 or 75 or 76 or 77 or 78 or 79 or 80 or 81 or 82 or 83 or 84 or 85 or 86 or 87 or 88 or 89 or 90 or 91 or 92 or 93 or 94 or 95 | 4218031 |
| 97 | substances of human origin.ab,ti. | 27 |
| 98 | SoHO.ab,ti. | 219 |
| 99 | donor-derived.ab,ti. | 5216 |
| 100 | human origin donations.ab,ti. | 0 |
| 101 | "cell* donor".ab,ti. | 1540 |
| 102 | "organ* donor".ab,ti. | 3107 |
| 103 | "blood donor*".ab,ti. | 29218 |
| 104 | blood components.ab,ti. | 8452 |
| 105 | "tissue* donor".ab,ti. | 312 |
| 106 | "cardiac don*".ab,ti. | 240 |
| 107 | "heart don*".ab,ti. | 745 |
| 108 | "liver don*".ab,ti. | 3102 |
| 109 | "cornea* don*".ab,ti. | 590 |
| 110 | "kidney don*".ab,ti. | 7418 |
| 111 | "pancreas don*".ab,ti. | 303 |
| 112 | "lung don*".ab,ti. | 708 |
| 113 | "intestine don*".ab,ti. | 19 |
| 114 | "vascularized composite allografts don*".ab,ti. | 0 |
| 115 | "VCAs don*".ab,ti. | 0 |
| 116 | risk mitigation.ab,ti. | 1335 |
| 117 | donor surveillance.ab,ti. | 24 |
| 118 | residual risks.ab,ti. | 212 |
| 119 | donor selection criteria.ab,ti. | 340 |
| 120 | donation material treatment.ab,ti. | 0 |
| 121 | donation material processing.ab,ti. | 0 |
| 122 | serious adverse reactions.ab,ti. | 1922 |
| 123 | blood safety.ab,ti. | 1976 |
| 124 | preventive measures.ab,ti. | 24447 |
| 125 | precautionary principle.ab,ti. | 914 |
| 126 | donor screening.ab,ti. | 1727 |
| 127 | laboratory screening.ab,ti. | 1266 |
| 128 | 97 or 98 or 99 or 100 or 101 or 102 or 103 or 104 or 105 or 106 or 107 or 108 or 109 or 110 or 111 or 112 or 113 or 114 or 115 or 116 or 117 or 118 or 119 or 120 or 121 or 122 or 123 or 124 or 125 or 126 or 127 | 90469 |
| 129 | 73 and 96 and 128 | 2400 |
| 130 | limit 129 to human | 2031 |

##

## **Web of Science searching algorithm for the fungal diseases searching**

SEARCHING ON TITLES

(mold OR mould OR yeasts OR entomophthoromycota OR Conidiobolus OR C. coronatus OR C. incongruus OR Basidiobolus ranarum OR Ascomycota OR B. dermatitidis OR Ajellomyces dermatitidis OR Ajellomyces capsulatus OR Paracoccidioides OR P. brasiliensis OR P. lutzii OR C. immitis OR C. posadasii OR Blastomyces OR Histoplasma OR Penicillium marneffei OR capsulatum OR farciminosum OR Lacazia OR Trichophyton OR Microsporum OR Epidermophyton OR Candida OR C. albicans OR C. glabrata OR C. parapsilosis OR C. tropicalis OR C. lusitaniae OR C. krusei OR C. guilliermondii OR Candidiasis OR candidemia OR C. auris OR C. dubliniensis OR C. kefyr OR C. rugosa OR Basidiomycota OR Cryptococcus OR C. neoformans OR C. gattii OR C. neoformans var. grubii OR Aspergillus OR A. fumigatus OR Aspergillosis OR A. flavus OR A. niger OR A. terreus OR A. versicolor OR A. calidoustus OR A. nidulans OR Blastomycosis OR Coccidioides OR coccidioidomycosis OR valley fever OR Histoplasmosis OR Mucormycosis OR Rhizopus OR Mucor OR Rhizomucor OR Syncephalastrum OR Cunninghamella bertholletiae OR Apophysomyces OR Lichtheimia OR Absidia OR Rhodotorula OR Saccharomyces OR S. cerevisiae OR Mycetoma OR Actinomycetoma OR Eumycetoma OR Pneumocystis jirovecii OR P. jirovecii OR P. carinii OR Sporothrix OR Sporotrichosis OR S. brasiliensis OR Paracoccidioidomycosis OR Talaromycosis OR Talaromyces marneffei OR vaginal yeast infection OR Pneumocystis pneumonia OR Non-Fumigatus Aspergilli OR Pseudoallescheria OR Malassezia furfur OR Fusarium OR Trichosporon asahii OR Scedosporium apiospermum OR Exserohilum rostratum OR E. rostratum OR Geotrichum OR Fungi OR Fungal)

AND

(transmission OR transmitt* OR transposal OR transmittable OR transferable OR transmissible OR contamination OR contaminating OR communicable OR contagious OR infect* OR harmful substances OR surgery OR transplantation OR blood transfusion OR stem cell transplant OR allogeneic transplant OR autologous transplant OR transplant recipients OR blood pack OR allograft recipients OR blood bank)

AND

(substances of human origin OR SoHO OR donor-derived OR human origin donations OR cell* donor OR organ* donor OR blood donor* OR blood components OR tissue* donor OR cardiac don* OR heart don* OR liver don* OR cornea* don* OR kidney don* OR pancreas don* OR lung don* OR intestine don* OR vascularized composite allografts don* OR VCAs don* OR risk mitigation OR donor surveillance OR residual risks OR donor selection criteria OR donation material treatment OR donation material processing OR serious adverse reactions OR blood safety OR preventive measures OR precautionary principle OR donor screening OR vaccin* OR laboratory screening)

##

## **Web of Science searching algorithm for the parasitic diseases searching**

SEARCHING ON TITLES

(parasites OR parasitic OR parasitism OR Trypanosomiasis OR T. brucei OR T. b. gambiense OR T. b. rhodesiense OR Entamoeba OR E. histolytica OR E. dispar OR Amebiasis OR Trypanosoma OR T. cruzi OR Babesia OR B. microti OR Babesiosis OR B. divergens OR B. duncani OR Balamuthia OR B. mandrillaris OR C. hepatica OR capillariasis OR Capillaria hepatica OR Chagas disease OR Pubic lice OR pubic crab OR Cystoisosporiasis OR Cystoisospora belli OR C. belli OR Isospora belli OR Pinworm OR E. vermicularis OR Enterobius vermicularis OR Giardia OR G. intestinalis OR G. lamblia OR G. duodenalis OR Giardiasis OR Leishmania OR Leishmaniasis OR Malaria OR Anopheles OR Plasmodium OR Microsporidiosis OR Anncaliia OR Encephalitozoon OR Microsporidium OR Nosema ocularum OR Pleistophora ronneafiei OR Trachipleistophora OR Tubulinosema acridophagus OR Vittaforma corneae OR Scabies OR Sarcoptes scabiei OR hominis OR enterobiasis OR Pneumocystis OR P. jirovecii OR P. carinii OR Strongyloidiasis OR Strongyloides OR S. stercoralis OR Toxoplasma OR Toxoplasmosis OR T. gondii OR Trichomoniasis OR Trichomonas vaginalis OR P. falciparum OR P. vivax OR P. malariae OR P. ovale OR P. knowlesi)

AND

(transmission OR transmitt* OR transposal OR transmittable OR transferable OR transmissible OR contamination OR contaminating OR communicable OR contagious OR infect* OR harmful substances OR surgery OR transplantation OR blood transfusion OR stem cell transplant OR allogeneic transplant OR autologous transplant OR transplant recipients OR blood pack OR allograft recipients OR blood bank)

AND

(substances of human origin OR SoHO OR donor-derived OR human origin donations OR cell* donor OR organ* donor OR blood donor* OR blood components OR tissue* donor OR cardiac don* OR heart don* OR liver don* OR cornea* don* OR kidney don* OR pancreas don* OR lung don* OR intestine don* OR vascularized composite allografts don* OR VCAs don* OR risk mitigation OR donor surveillance OR residual risks OR donor selection criteria OR donation material treatment OR donation material processing OR serious adverse reactions OR blood safety OR preventive measures OR precautionary principle OR donor screening OR vaccin* OR laboratory screening)

## **Scopus searching algorithm for the fungal diseases searching**

SEARCHING ON TITLES (Limit to human, article, conference paper and short survey) and (journals, conference proceedings and reports)

(mold OR mould OR yeasts OR entomophthoromycota OR Conidiobolus OR “C. coronatus” OR “C. incongruous” OR “Basidiobolus ranarum” OR Ascomycota OR “B. dermatitidis” OR “Ajellomyces dermatitidis” OR “Ajellomyces capsulatus” OR Paracoccidioides OR “P. brasiliensis” OR “P. lutzii” OR “C. immitis” OR “C. posadasii” OR Blastomyces OR Histoplasma OR “Penicillium marneffei” OR capsulatum OR farciminosum OR Lacazia OR Trichophyton OR Microsporum OR Epidermophyton OR Candida OR “C. albicans” OR “C. glabrata” OR “C. parapsilosis” OR “C. tropicalis” OR “C. lusitaniae” OR “C. krusei” OR “C. guilliermondii” OR Candidiasis OR candidemia OR “C. auris” OR “C. dubliniensis” OR “C. kefyr” OR “C. rugosa” OR Basidiomycota OR Cryptococcus OR “C. neoformans” OR “C. gattii” OR “C. neoformans var. grubii” OR Aspergillus OR “A. fumigatus” OR Aspergillosis OR “A. flavus” OR “A. niger” OR “A. terreus” OR “A. versicolor” OR “A. calidoustus” OR “A. nidulans” OR Blastomycosis OR Coccidioides OR coccidioidomycosis OR “valley fever” OR Histoplasmosis OR Mucormycosis OR Rhizopus OR Mucor OR Rhizomucor OR Syncephalastrum OR “Cunninghamella bertholletiae” OR Apophysomyces OR Lichtheimia OR Absidia OR Rhodotorula OR Saccharomyces OR “S. cerevisiae” OR Mycetoma OR Actinomycetoma OR Eumycetoma OR “Pneumocystis jirovecii” OR “P. jirovecii” OR “P. carinii” OR Sporothrix OR Sporotrichosis OR “S. brasiliensis” OR Paracoccidioidomycosis OR Talaromycosis OR “Talaromyces marneffei” OR “vaginal yeast infection” OR “Pneumocystis pneumonia” OR “Non-Fumigatus Aspergilli” OR Pseudoallescheria OR “Malassezia furfur” OR Fusarium OR “Trichosporon asahii” OR “Scedosporium apiospermum” OR “Exserohilum rostratum” OR “E. rostratum” OR Geotrichum OR Fungi OR Fungal)

AND

(transmission OR transmitt* OR transposal OR transmittable OR transferable OR transmissible OR contamination OR contaminating OR communicable OR contagious OR infect* OR harmful substances OR surgery OR transplantation OR “blood transfusion” OR “stem cell transplant” OR “allogeneic transplant” OR “autologous transplant” OR “transplant recipients” OR “blood pack” OR “allograft recipients” OR “blood bank”)

AND

(“substances of human origin” OR SoHO OR “donor-derived” OR “human origin donations” OR “cell* donor” OR “organ* donor” OR “blood donor*” OR “blood components” OR “tissue* donor” OR “cardiac don*” OR “heart don*” OR “liver don*” OR “cornea* don*” OR “kidney don*” OR “pancreas don*” OR “lung don*” OR “intestine don*” OR “vascularized composite allografts don*” OR “VCAs don*” OR “risk mitigation” OR “donor surveillance” OR “residual risks” OR “donor selection criteria” OR “donation material treatment” OR “donation material processing” OR “serious adverse reactions” OR “blood safety” OR “preventive measures” OR “precautionary principle” OR “donor screening” OR vaccin* OR “laboratory screening”)

## **Scopus searching algorithm for the parasitic diseases searching**

SEARCHING ON TITLES (Limit to humans article, conference paper) and (journals, conference proceedings)

(parasites OR parasitic OR parasitism OR Trypanosomiasis OR “T. brucei” OR “T. b. gambiense” OR “T. b. rhodesiense” OR Entamoeba OR “E. histolytica” OR “E. dispar” OR Amebiasis OR Trypanosoma OR “T. cruzi” OR Babesia OR “B. microti” OR Babesiosis OR “B. divergens” OR “B. duncani” OR Balamuthia OR “B. mandrillaris” OR “C. hepatica” OR capillariasis OR “Capillaria hepatica” OR “Chagas disease” OR “Pubic lice” OR “pubic crab” OR Cystoisosporiasis OR “Cystoisospora belli” OR “C. belli” OR “Isospora belli” OR Pinworm OR “E. vermicularis” OR “Enterobius vermicularis” OR Giardia OR “G. intestinalis” OR “G. lamblia” OR “G. duodenalis” OR Giardiasis OR Leishmania OR Leishmaniasis OR Malaria OR Anopheles OR Plasmodium OR Microsporidiosis OR Anncaliia OR Encephalitozoon OR Microsporidium OR “Nosema ocularum” OR “Pleistophora ronneafiei” OR Trachipleistophora OR “Tubulinosema acridophagus” OR “Vittaforma corneae” OR Scabies OR “Sarcoptes scabiei” OR hominis OR enterobiasis OR Pneumocystis OR “P. jirovecii” OR “P. carinii” OR Strongyloidiasis OR Strongyloides OR “S. stercoralis” OR Toxoplasma OR Toxoplasmosis OR “T. gondii” OR Trichomoniasis OR “Trichomonas vaginalis” OR “P. falciparum” OR “P. vivax” OR “P. malariae” OR “P. ovale” OR “P. knowlesi”)

AND

(transmission OR transmitt* OR transposal OR transmittable OR transferable OR transmissible OR contamination OR contaminating OR communicable OR contagious OR infect* OR harmful substances OR surgery OR transplantation OR “blood transfusion” OR “stem cell transplant” OR “allogeneic transplant” OR “autologous transplant” OR “transplant recipients” OR “blood pack” OR “allograft recipients” OR “blood bank”)

AND

(“substances of human origin” OR SoHO OR “donor-derived” OR “human origin donations” OR “cell* donor” OR “organ* donor” OR “blood donor*” OR “blood components” OR “tissue* donor” OR “cardiac don*” OR “heart don*” OR “liver don*” OR “cornea* don*” OR “kidney don*” OR “pancreas don*” OR “lung don*” OR “intestine don*” OR “vascularized composite allografts don*” OR “VCAs don*” OR “risk mitigation” OR “donor surveillance” OR “residual risks” OR “donor selection criteria” OR “donation material treatment” OR “donation material processing” OR “serious adverse reactions” OR “blood safety” OR “preventive measures” OR “precautionary principle” OR “donor screening” OR vaccin* OR “laboratory screening”)

##

## **Cochrane library searching algorithm for the fungal diseases searching**

|  | Search | Hits |
| --- | --- | --- |
| #1 | ((mold OR mould OR yeasts OR entomophthoromycota OR Conidiobolus OR C. coronatus OR C. incongruus OR Basidiobolus ranarum OR Ascomycota OR B. dermatitidis OR Ajellomyces dermatitidis OR Ajellomyces capsulatus OR Paracoccidioides OR P. brasiliensis OR P. lutzii OR C. immitis OR C. posadasii OR Blastomyces OR Histoplasma OR Penicillium marneffei OR capsulatum OR farciminosum OR Lacazia OR Trichophyton OR Microsporum OR Epidermophyton OR Candida OR C. albicans OR C. glabrata OR C. parapsilosis OR C. tropicalis OR C. lusitaniae OR C. krusei OR C. guilliermondii OR Candidiasis OR candidemia OR C. auris OR C. dubliniensis OR C. kefyr OR C. rugosa OR Basidiomycota OR Cryptococcus OR C. neoformans OR C. gattii OR C. neoformans var. grubii OR Aspergillus OR A. fumigatus OR Aspergillosis OR A. flavus OR A. niger OR A. terreus OR A. versicolor OR A. calidoustus OR A. nidulans OR Blastomycosis OR Coccidioides OR coccidioidomycosis OR valley fever OR Histoplasmosis OR Mucormycosis OR Rhizopus OR Mucor OR Rhizomucor OR Syncephalastrum OR Cunninghamella bertholletiae OR Apophysomyces OR Lichtheimia OR Absidia OR Rhodotorula OR Saccharomyces OR S. cerevisiae OR Mycetoma OR Actinomycetoma OR Eumycetoma OR Pneumocystis jirovecii OR P. jirovecii OR P. carinii OR Sporothrix OR Sporotrichosis OR S. brasiliensis OR Paracoccidioidomycosis OR Talaromycosis OR Talaromyces marneffei OR vaginal yeast infection OR Pneumocystis pneumonia OR Non-Fumigatus Aspergilli OR Pseudoallescheria OR Malassezia furfur OR Fusarium OR Trichosporon asahii OR Scedosporium apiospermum OR Exserohilum rostratum OR E. rostratum OR Geotrichum OR Fungi OR Fungal)):ti,ab,kw in Trials (Word variations have been searched) | 9417 |
| #2 | ((transmission OR transmitt* OR transposal OR transmittable OR transferable OR transmissible OR contamination OR contaminating OR communicable OR contagious OR infect* OR harmful substances OR surgery OR transplantation OR blood transfusion OR stem cell transplant OR allogeneic transplant OR autologous transplant OR transplant recipients OR blood pack OR allograft recipients OR blood bank)):ti,ab,kw in Trials (Word variations have been searched) | 365391 |
| #3 | ((substances of human origin OR SoHO OR donor-derived OR human origin donations OR cell* donor OR organ* donor OR blood donor* OR blood components OR tissue* donor OR cardiac don* OR heart don* OR liver don* OR cornea* don* OR kidney don* OR pancreas don* OR lung don* OR intestine don* OR vascularized composite allografts don* OR VCAs don* OR risk mitigation OR donor surveillance OR residual risks OR donor selection criteria OR donation material treatment OR donation material processing OR serious adverse reactions OR blood safety OR preventive measures OR precautionary principle OR donor screening OR vaccin* OR laboratory screening)):ti,ab,kw in Trials (Word variations have been searched) | 167923 |
| #4 | #1 AND #2 AND #3 | 1098 |

## **Cochrane library searching algorithm for the parasitic diseases searching**

|  | Search | Hits |
| --- | --- | --- |
| #1 | ((parasites OR parasitic OR parasitism OR Trypanosomiasis OR T. brucei OR T. b. gambiense OR T. b. rhodesiense OR Entamoeba OR E. histolytica OR E. dispar OR Amebiasis OR Trypanosoma OR T. cruzi OR Babesia OR B. microti OR Babesiosis OR B. divergens OR B. duncani OR Balamuthia OR B. mandrillaris OR C. hepatica OR capillariasis OR Capillaria hepatica OR Chagas disease OR Pubic lice OR pubic crab OR Cystoisosporiasis OR Cystoisospora belli OR C. belli OR Isospora belli OR Pinworm OR E. vermicularis OR Enterobius vermicularis OR Giardia OR G. intestinalis OR G. lamblia OR G. duodenalis OR Giardiasis OR Leishmania OR Leishmaniasis OR Malaria OR Anopheles OR Plasmodium OR Microsporidiosis OR Anncaliia OR Encephalitozoon OR Microsporidium OR Nosema ocularum OR Pleistophora ronneafiei OR Trachipleistophora OR Tubulinosema acridophagus OR Vittaforma corneae OR Scabies OR Sarcoptes scabiei OR hominis OR enterobiasis OR Pneumocystis OR P. jirovecii OR P. carinii OR Strongyloidiasis OR Strongyloides OR S. stercoralis OR Toxoplasma OR Toxoplasmosis OR T. gondii OR Trichomoniasis OR Trichomonas vaginalis OR P. falciparum OR P. vivax OR P. malariae OR P. ovale OR P. knowlesi)):ti,ab,kw in Trials (Word variations have been searched) | 11126 |
| #2 | ((transmission OR transmitt* OR transposal OR transmittable OR transferable OR transmissible OR contamination OR contaminating OR communicable OR contagious OR infect* OR harmful substances OR surgery OR transplantation OR blood transfusion OR stem cell transplant OR allogeneic transplant OR autologous transplant OR transplant recipients OR blood pack OR allograft recipients OR blood bank)):ti,ab,kw in Trials (Word variations have been searched) | 365391 |
| #3 | ((substances of human origin OR SoHO OR donor-derived OR human origin donations OR cell* donor OR organ* donor OR blood donor* OR blood components OR tissue* donor OR cardiac don* OR heart don* OR liver don* OR cornea* don* OR kidney don* OR pancreas don* OR lung don* OR intestine don* OR vascularized composite allografts don* OR VCAs don* OR risk mitigation OR donor surveillance OR residual risks OR donor selection criteria OR donation material treatment OR donation material processing OR serious adverse reactions OR blood safety OR preventive measures OR precautionary principle OR donor screening OR vaccin* OR laboratory screening)):ti,ab,kw in Trials (Word variations have been searched) | 167923 |
| #4 | #1 AND #2 AND #3 | 1572 |

## **CINAHL searching algorithm for the fungal diseases searching**

| **#** | **Query** | **Results** |
| --- | --- | --- |
| S10 | S3 AND S6 AND S9 | 128 |
| S9 | S7 OR S8 | 63,951 |
| S8 | AB (substances of human origin OR SoHO OR donor-derived OR human origin donations OR cell* donor OR organ* donor OR blood donor* OR blood components OR tissue* donor OR cardiac don* OR heart don* OR liver don* OR cornea* don* OR kidney don* OR pancreas don* OR lung don* OR intestine don* OR vascularized composite allografts don* OR VCAs don* OR risk mitigation OR donor surveillance OR residual risks OR donor selection criteria OR donation material treatment OR donation material processing OR serious adverse reactions OR blood safety OR preventive measures OR precautionary principle OR donor screening OR vaccin* OR laboratory screening) | 45,863 |
| S7 | TI (substances of human origin OR SoHO OR donor-derived OR human origin donations OR cell* donor OR organ* donor OR blood donor* OR blood components OR tissue* donor OR cardiac don* OR heart don* OR liver don* OR cornea* don* OR kidney don* OR pancreas don* OR lung don* OR intestine don* OR vascularized composite allografts don* OR VCAs don* OR risk mitigation OR donor surveillance OR residual risks OR donor selection criteria OR donation material treatment OR donation material processing OR serious adverse reactions OR blood safety OR preventive measures OR precautionary principle OR donor screening OR vaccin* OR laboratory screening) | 33,255 |
| S6 | S4 OR S5 | 208,538 |
| S5 | AU (transmission OR transmitt* OR transposal OR transmittable OR transferable OR transmissible OR contamination OR contaminating OR communicable OR contagious OR infect* OR harmful substances OR surgery OR transplantation OR blood transfusion OR stem cell transplant OR allogeneic transplant OR autologous transplant OR transplant recipients OR blood pack OR allograft recipients OR blood bank) | 189 |
| S4 | TI (transmission OR transmitt* OR transposal OR transmittable OR transferable OR transmissible OR contamination OR contaminating OR communicable OR contagious OR infect* OR harmful substances OR surgery OR transplantation OR blood transfusion OR stem cell transplant OR allogeneic transplant OR autologous transplant OR transplant recipients OR blood pack OR allograft recipients OR blood bank) | 208,395 |
| S3 | S1 OR S2 | 21,316 |
| S2 | AB (mold OR mould OR yeasts OR entomophthoromycota OR Conidiobolus OR C. coronatus OR C. incongruus OR Basidiobolus ranarum OR Ascomycota OR B. dermatitidis OR Ajellomyces dermatitidis OR Ajellomyces capsulatus OR Paracoccidioides OR P. brasiliensis OR P. lutzii OR C. immitis OR C. posadasii OR Blastomyces OR Histoplasma OR Penicillium marneffei OR capsulatum OR farciminosum OR Lacazia OR Trichophyton OR Microsporum OR Epidermophyton OR Candida OR C. albicans OR C. glabrata OR C. parapsilosis OR C. tropicalis OR C. lusitaniae OR C. krusei OR C. guilliermondii OR Candidiasis OR candidemia OR C. auris OR C. dubliniensis OR C. kefyr OR C. rugosa OR Basidiomycota OR Cryptococcus OR C. neoformans OR C. gattii OR C. neoformans var. grubii OR Aspergillus OR A. fumigatus OR Aspergillosis OR A. flavus OR A. niger OR A. terreus OR A. versicolor OR A. calidoustus OR A. nidulans OR Blastomycosis OR Coccidioides OR coccidioidomycosis OR valley fever OR Histoplasmosis OR Mucormycosis OR Rhizopus OR Mucor OR Rhizomucor OR Syncephalastrum OR Cunninghamella bertholletiae OR Apophysomyces OR Lichtheimia OR Absidia OR Rhodotorula OR Saccharomyces OR S. cerevisiae OR Mycetoma OR Actinomycetoma OR Eumycetoma OR Pneumocystis jirovecii OR P. jirovecii OR P. carinii OR Sporothrix OR Sporotrichosis OR S. brasiliensis OR Paracoccidioidomycosis OR Talaromycosis OR Talaromyces marneffei OR vaginal yeast infection OR Pneumocystis pneumonia OR Non- Fumigatus Aspergilli OR Pseudoallescheria OR Malassezia furfur OR Fusarium OR Trichosporon asahii OR Scedosporium apiospermum OR Exserohilum rostratum OR E. rostratum OR Geotrichum OR Fungi OR Fungal) | 16,587 |
| S1 | TI (mold OR mould OR yeasts OR entomophthoromycota OR Conidiobolus OR C. coronatus OR C. incongruus OR Basidiobolus ranarum OR Ascomycota OR B. dermatitidis OR Ajellomyces dermatitidis OR Ajellomyces capsulatus OR Paracoccidioides OR P. brasiliensis OR P. lutzii OR C. immitis OR C. posadasii OR Blastomyces OR Histoplasma OR Penicillium marneffei OR capsulatum OR farciminosum OR Lacazia OR Trichophyton OR Microsporum OR Epidermophyton OR Candida OR C. albicans OR C. glabrata OR C. parapsilosis OR C. tropicalis OR C. lusitaniae OR C. krusei OR C. guilliermondii OR Candidiasis OR candidemia OR C. auris OR C. dubliniensis OR C. kefyr OR C. rugosa OR Basidiomycota OR Cryptococcus OR C. neoformans OR C. gattii OR C. neoformans var. grubii OR Aspergillus OR A. fumigatus OR Aspergillosis OR A. flavus OR A. niger OR A. terreus OR A. versicolor OR A. calidoustus OR A. nidulans OR Blastomycosis OR Coccidioides OR coccidioidomycosis OR valley fever OR Histoplasmosis OR Mucormycosis OR Rhizopus OR Mucor OR Rhizomucor OR Syncephalastrum OR Cunninghamella bertholletiae OR Apophysomyces OR Lichtheimia OR Absidia OR Rhodotorula OR Saccharomyces OR S. cerevisiae OR Mycetoma OR Actinomycetoma OR Eumycetoma OR Pneumocystis jirovecii OR P. jirovecii OR P. carinii OR Sporothrix OR Sporotrichosis OR S. brasiliensis OR Paracoccidioidomycosis OR Talaromycosis OR Talaromyces marneffei OR vaginal yeast infection OR Pneumocystis pneumonia OR Non- Fumigatus Aspergilli OR Pseudoallescheria OR Malassezia furfur OR Fusarium OR Trichosporon asahii OR Scedosporium apiospermum OR Exserohilum rostratum OR E. rostratum OR Geotrichum OR Fungi OR Fungal) | 10,732 |

## **CINAHL searching algorithm for the parasitic diseases searching**

| **#** | **Query** | **Results** |
| --- | --- | --- |
| S10 | S3 AND S6 AND S9 | 780 |
| S9 | S7 OR S8 | 63,951 |
| S8 | AB (substances of human origin OR SoHO OR donor-derived OR human origin donations OR cell* donor OR organ* donor OR blood donor* OR blood components OR tissue* donor OR cardiac don* OR heart don* OR liver don* OR cornea* don* OR kidney don* OR pancreas don* OR lung don* OR intestine don* OR vascularized composite allografts don* OR VCAs don* OR risk mitigation OR donor surveillance OR residual risks OR donor selection criteria OR donation material treatment OR donation material processing OR serious adverse reactions OR blood safety OR preventive measures OR precautionary principle OR donor screening OR vaccin* OR laboratory screening) | 45,863 |
| S7 | TI (substances of human origin OR SoHO OR donor-derived OR human origin donations OR cell* donor OR organ* donor OR blood donor* OR blood components OR tissue* donor OR cardiac don* OR heart don* OR liver don* OR cornea* don* OR kidney don* OR pancreas don* OR lung don* OR intestine don* OR vascularized composite allografts don* OR VCAs don* OR risk mitigation OR donor surveillance OR residual risks OR donor selection criteria OR donation material treatment OR donation material processing OR serious adverse reactions OR blood safety OR preventive measures OR precautionary principle OR donor screening OR vaccin* OR laboratory screening) | 33,255 |
| S6 | S4 OR S5 | 489,201 |
| S5 | AU (transmission OR transmitt* OR transposal OR transmittable OR transferable OR transmissible OR contamination OR contaminating OR communicable OR contagious OR infect* OR harmful substances OR surgery OR transplantation OR blood transfusion OR stem cell transplant OR allogeneic transplant OR autologous transplant OR transplant recipients OR blood pack OR allograft recipients OR blood bank) | 383,359 |
| S4 | TI (transmission OR transmitt* OR transposal OR transmittable OR transferable OR transmissible OR contamination OR contaminating OR communicable OR contagious OR infect* OR harmful substances OR surgery OR transplantation OR blood transfusion OR stem cell transplant OR allogeneic transplant OR autologous transplant OR transplant recipients OR blood pack OR allograft recipients OR blood bank) | 208,395 |
| S3 | S1 OR S2 | 20,670 |
| S2 | AB (parasites OR parasitic OR parasitism OR Trypanosomiasis OR T. brucei OR T. b. gambiense OR T. b. rhodesiense OR Entamoeba OR E. histolytica OR E. dispar OR Amebiasis OR Trypanosoma OR T. cruzi OR Babesia OR B. microti OR Babesiosis OR B. divergens OR B. duncani OR Balamuthia OR B. mandrillaris OR C. hepatica OR capillariasis OR Capillaria hepatica OR Chagas disease OR Pubic lice OR pubic crab OR Cystoisosporiasis OR Cystoisospora belli OR C. belli OR Isospora belli OR Pinworm OR E. vermicularis OR Enterobius vermicularis OR Giardia OR G. intestinalis OR G. lamblia OR G. duodenalis OR Giardiasis OR Leishmania OR Leishmaniasis OR Malaria OR Anopheles OR Plasmodium OR Microsporidiosis OR Anncaliia OR Encephalitozoon OR Microsporidium OR Nosema ocularum OR Pleistophora ronneafiei OR Trachipleistophora OR Tubulinosema acridophagus OR Vittaforma corneae OR Scabies OR Sarcoptes scabiei OR hominis OR enterobiasis OR Pneumocystis OR P. jirovecii OR P. carinii OR Strongyloidiasis OR Strongyloides OR S. stercoralis OR Toxoplasma OR Toxoplasmosis OR T. gondii OR Trichomoniasis OR Trichomonas vaginalis OR P. falciparum OR P. vivax OR P. malariae OR P. ovale OR P. knowlesi) | 14,521 |
| S1 | TI (parasites OR parasitic OR parasitism OR Trypanosomiasis OR T. brucei OR T. b. gambiense OR T. b. rhodesiense OR Entamoeba OR E. histolytica OR E. dispar OR Amebiasis OR Trypanosoma OR T. cruzi OR Babesia OR B. microti OR Babesiosis OR B. divergens OR B. duncani OR Balamuthia OR B. mandrillaris OR C. hepatica OR capillariasis OR Capillaria hepatica OR Chagas disease OR Pubic lice OR pubic crab OR Cystoisosporiasis OR Cystoisospora belli OR C. belli OR Isospora belli OR Pinworm OR E. vermicularis OR Enterobius vermicularis OR Giardia OR G. intestinalis OR G. lamblia OR G. duodenalis OR Giardiasis OR Leishmania OR Leishmaniasis OR Malaria OR Anopheles OR Plasmodium OR Microsporidiosis OR Anncaliia OR Encephalitozoon OR Microsporidium OR Nosema ocularum OR Pleistophora ronneafiei OR Trachipleistophora OR Tubulinosema acridophagus OR Vittaforma corneae OR Scabies OR Sarcoptes scabiei OR hominis OR enterobiasis OR Pneumocystis OR P. jirovecii OR P. carinii OR Strongyloidiasis OR Strongyloides OR S. stercoralis OR Toxoplasma OR Toxoplasmosis OR T. gondii OR Trichomoniasis OR Trichomonas vaginalis OR P. falciparum OR P. vivax OR P. malariae OR P. ovale OR P. knowlesi) | 13,011 |
